# Lactoferrin suppresses the progression of colon cancer under hyperglycemia by targeting *WTAP*/m^6^A/*NT5DC3*/*HKDC1* axis

**DOI:** 10.1186/s12967-023-03983-1

**Published:** 2023-02-28

**Authors:** Huiying Li, Chaonan Li, Boyang Zhang, Hongpeng Jiang

**Affiliations:** 1 College of Biological Sciences and Technology, Beijing Key Laboratory of Food Processing and Safety in Forestry, Beijing Forestry University, Beijing, 100083 People’s Republic of China; 2 Department of Nutrition and Health, China Agricultural University, Beijing, 100083 People’s Republic of China; 3 Department of General Surgery, Beijing Key Laboratory of Cancer Invasion and Metastasis Research and National Clinical Research Center for Digestive Diseases, Beijing Friendship Hospital, Capital Medical University, Beijing, 100050 People’s Republic of China

**Keywords:** Type 2 diabetes (T2D), Colon cancer, NT5DC3, Lactoferrin (LF), RNA m^6^A

## Abstract

**Background:**

Although the relationship between type 2 diabetes (T2D) and the increased risk of colorectal carcinogenesis is widely defined in clinical studies, the therapeutic methods and molecular mechanism of T2D-induced colon cancer and how does hyperglycemia affect the progression is still unknown. Here, we studied the function of lactoferrin (LF) in suppressing the progression of colon cancer in T2D mice, and uncovered the related molecular mechanisms in DNA 5mC and RNA m6A levels.

**Methods:**

We examined the effects of LF (50% iron saturation) on the migration and invasion of colon tumor cells under high concentration of glucose. Then, transcriptomics and DNA methylation profilings of colon tumor cells was co-analyzed to screen out the special gene (*NT5DC3*), and the expression level of NT5DC3 in 75 clinical blood samples was detected by q-PCR and western blot, to investigate whether NT5DC3 was a biomarker to distinguish T2D patients and T2D-induced colon cancer patients from healthy volunteers. Futhermore, in T2D mouse with xenografted colon tumor models, the inhibitory effects of LF and NT5DC3 protein on colon tumors were investigated. In addition, epigenetic alterations were measured to examine the 5mC/m^6^A modification sites of *NT5DC3* regulated by LF. Utilizing siRNA fragments of eight m^6^A-related genes, the special gene (*WTAP*) regulating m^6^A of *NT5DC* was proved, and the effect of LF on *WTAP*/*NT5DC3*/*HKDC1* axis was finally evaluated.

**Results:**

A special gene *NT5DC3* was screened out through co-analysis of transcriptomics and DNA methylation profiling, and *HKDC1* might be a downstream sensor of *NT5DC3*. Mechanistically, LF-dependent cellular DNA 5mC and RNA m^6^A profiling remodeling transcriptionally regulate NT5DC3 expression. *WTAP* plays a key role in regulating *NT5DC3* m^6^A modification and subsequently controls *NT5DC3* downstream target *HKDC1* expression. Moreover, co-treatment of lactoferrin and NT5DC3 protein restrains the growth of colon tumors by altering the aberrant epigenetic markers. Strikingly, clinical blood samples analysis demonstrates NT5DC3 protein expression is required to direct the distinction of T2D or T2D-induced colon cancer with healthy humans.

**Conclusions:**

Together, this study reveals that lactoferrin acts as a major factor to repress the progression of colon cancer under hyperglycemia, thus, significantly expanding the landscape of natural dietary mediated tumor suppression.

**Graphical Abstract:**

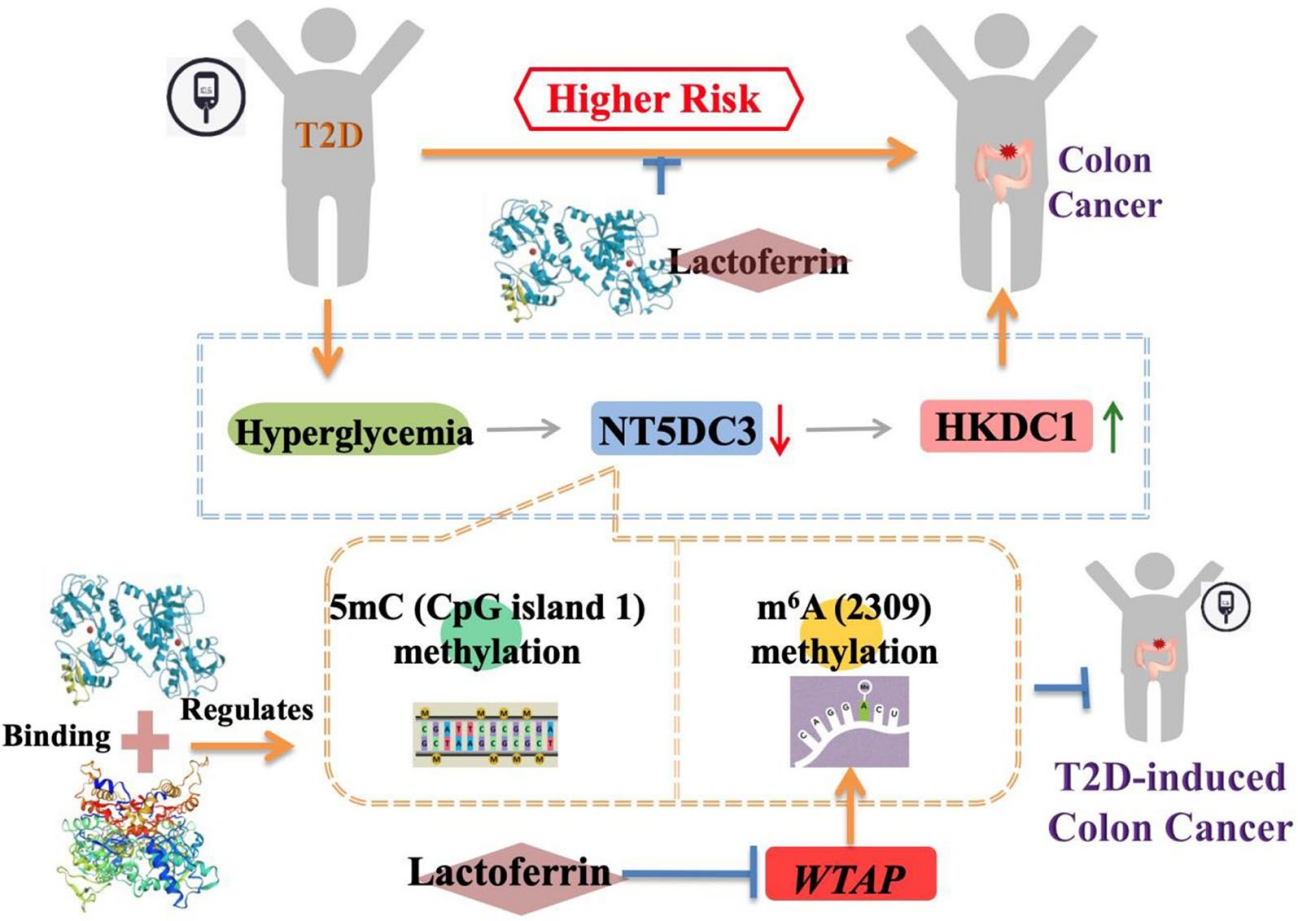

**Supplementary Information:**

The online version contains supplementary material available at 10.1186/s12967-023-03983-1.

## Introduction

Epidemiological and clinical evidence suggests that type 2 diabetes (T2D) is linked to an increased risk of cancer, especially colon cancer [1–4]. Several meta-analyses reveal that the patients with T2D have up to 40% higher risks of suffering bladder, breast, colorectal, and kidney cancers than the patients without T2D [1–4]. Interestingly, research is shedding light on the potential biological links between these two diseases, with some studies suggesting that hyperglycemia might be one of the possible reasons directly linking diabetes and cancer [5]. Hyperglycemic conditions directly reprogram the epigenome, in which DNA hydroxymethylation and protein phosphorylation are mediated as a key switch and regulatory pathway that connects diabetes to cancer [6]. DNA methylation and hydroxymethylation are critical epigenetic processes involved in regulating the expression of tumor suppressor genes, as well as other cancer-related genes that are frequently perturbed in tumor cells [7]. In addition to cancer, alterations in the DNA methylation profile also have been identified in T2D patients [8]. Furthermore, as the most abundant and reversible modification happens at RNA, N^6^-methyladenosine (m^6^A) emerges as a new type of epigenetic regulation in recent years. A number of studies have shown that aberrancies in m^6^A are associated with both cancer and diabetes, and in several diabetic patients, the mRNA level of *METTL3* is proved to increase when compared with the healthy volunteers [9]. The integration of DNA/RNA methylation data has further elucidated the pathways of cancer signaling networks [10–12]. A wholistic insight that highlights and prioritizes DNA 5mC/RNA m^6^A is required to further unravel the biological complexity of the relationship between T2D and colon cancer.

The coexistence of diabetes and cancer has been found to increase individual mortality, yet some cancer therapeutics can result in transient or permanent damage [13, 14]. The targeting therapies of hormone-based drugs (such as glucocorticoids), tyrosine kinase inhibitors (nilotinib and pazopanib), and mTOR inhibitors (everolimus and temsirolimus), for instance, have been connected to the development of high rates of hyperglycemia. However, antidiabetic therapies, such as metformin, also show implications as targets for the treatment of cancer [15]. Hence, additional studies should be performed to optimize cancer-targeting therapy strategies in patients with diabetes. There is currently increasing interest in the use of natural compounds (diet therapy) to address this issue. One prospective cohort study, based on 136,384 individuals in 21 countries on five continents, reports the association of dairy consumption with a lower risk of mortality [16], and an increased consumption of dairy products has also been linked to a significant reduction in colon cancer, and the relative risk of T2D is reportedly nearly 10% lower in people with a high milk intake [17]. Thus, whether special bioactive components in dairy products play roles in the progression of T2D-colon cancer, deserves our further research.

Lactoferrin (LF), one of the active biological factors in milk, has recently been considered as a nutraceutical protein [18]. Due to the two Fe^3 +^ binding sites in its structure, lactoferrin can be divided into the apo-type (without iron atom), a single-type (binding with 1 iron atom), and the holo-type (binding with 2 iron atoms), which have been proved to be tightly related to its bioactivities including anti-inflammation, anti-oxidation, anti-virus and anti-tumor [19–21]. LF is shown to improve the insulin-signaling response, used as an unexpected application in a potential treatment for diabetes [22]. Studies using both in vitro and in vivo models have verified that LF has beneficial effects in cancer treatment, therein suggesting its anti-tumor properties [23]. Recently, the antitumor activities of LF were proved in a human colon tumor model and HT29 tumor-bearing nude mice via the inhibition of tumor angiogenesis and metastasis signal pathways [20]. However, there were few studies that proved lactoferrin with 50% iron saturation suppressed the progression of colon cancer under a high concentration of glucose. Here, we demonstrate the potential advantage of LF (with 50% iron saturation) in the suppression of colon cancer in T2D mice. This study reveals the underlying mechanism by which LF-dependent DNA 5mC and RNA m^6^A remodeling in epigenetically regulating the NT5DC3/HKDC1 axis, and subsequently inhibiting colon cancer progression under hyperglycemia.

## Results

### Development of colon cancer in T2D mice model

**Fig. 1:**
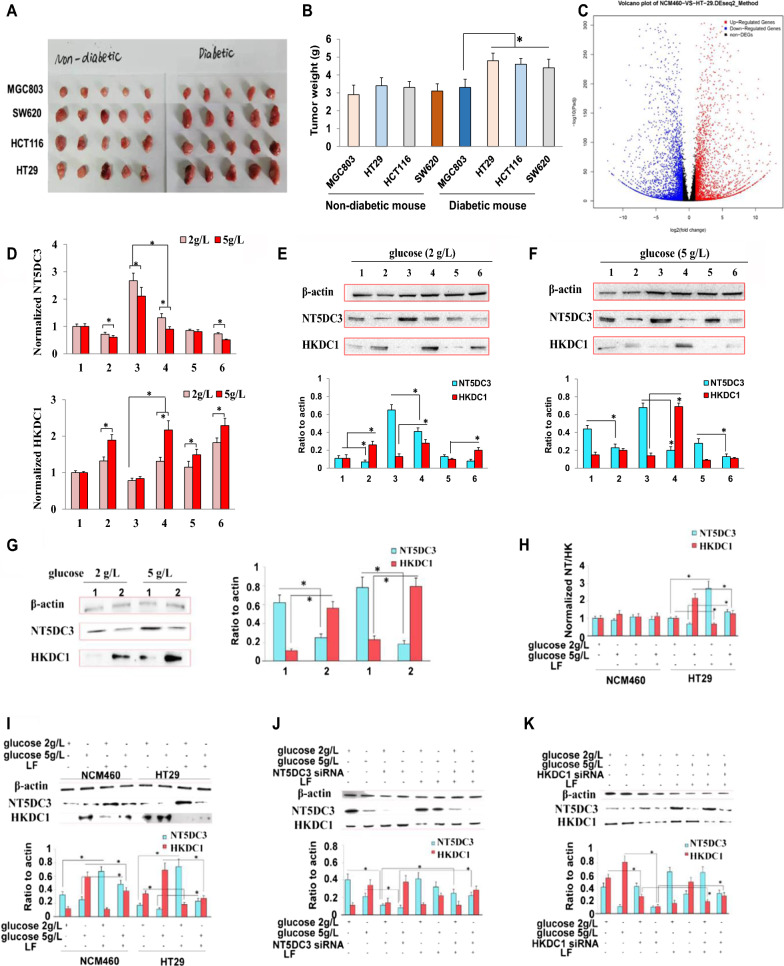
The effect of LF in regulating NT5DC3 and HKDC1. **A** Comparison of four xenografted tumors dissected from non-diabetic and diabetic mice in the BALB/c nude mouse model. **B** Average tumor weight in non-diabetic and diabetic mice implanted with four types of cancer cells. Data are presented as mean ± SD, * *P* < 0.05 compared with the HT29 group (n = 5). The corresponding mean weight in HT29 and HCT116 groups exceeded the others as well. **C** Volcano plot of identified DEGs. A total of 5,585 differentially expressed genes (DEGs, *P* value < 0.01, and with a fold change < 0.5 or > 2) were identified among HT29 colon cancer cells and NCM460 normal human colon cells, of which 2,534 were up-regulated, while 3,051 were down-regulated (Supplementary Transcriptomics analysis data in Data and Code Availability section). **D** Normalized mRNA levels of *NT5DC3* and *HKDC1* in HT29 cells treated with normal-glucose (2 g·L^−1^) or high-glucose (5 g·L^−1^) medium, in 6 types of cells; 1–6 indicated GES1 cells, MGC803 cells, NCM460 cells, HT29 cells, HK2 cells, and SW839 cells, respectively, * *P* < 0.05 (n = 3). **E** Protein levels of NT5DC3 and HKDC1 in 6 types of cells under normal glucose, * *P* < 0.05 (n = 3). **F** Protein levels of NT5DC3 and HKDC1 in 6 types of cells under a high glucose condition, * *P* < 0.05 (n = 3). **G** Protein levels of NT5DC3 and HKDC1 in HT29 cells under normal glucose and high glucose, * *P* < 0.05 (n = 3). **H** Normalized mRNA levels of *NT5DC3* and *HKDC1* under normal glucose (2 g·L^−1^) or high glucose (5 g·L^−1^) in NCM460 cells and HT29 cells, * *P* < 0.05 (n = 3). **I** NT5DC3 and HKDC1 protein levels under normal glucose (2 g·L^−1^) or high glucose (5 g·L^−1^), in NCM460 cells and HT29 cells, * *P* < 0.05 (n = 3). **J** Protein expressions of NT5DC3 and HKDC1 in HT29 cells treated with *NT5DC3* siRNA, *P < 0.05 (n = 3). **K** Protein expressions of NT5DC3 and HKDC1 in HT29 cells treated with *HKDC1* siRNA, *P < 0.05 (n = 3)

To set up the in vivo colon tumor model in T2D mice, we conducted a xenograft model screen by using cancer cells derived from different organs, including gastric cancer cell line MGC803, colon cancer cell lines HT29, HCT116, and SW620. The tumor formation time and tumor weight in both T2D and non-diabetic BALB/c nude mice were determined. Though the diabetic mice were found to be at a comparatively higher risk of developing tumors, there were significant differences in the tumor formation: the tumor formation time for HT29 cells (17.2 ± 1.4 d in normal mice and 11.4 ± 1.5 d in diabetic mice) and HCT116 cells (18.4 ± 1.7 d in normal mice and 13.8 ± 1.8 d in diabetic mice) was significantly shorter than other cell lines (MGC803: 20.6 ± 2.1 d in normal mice and 16.8 ± 2.3 d in diabetic mice; SW620: 19.0 ± 2.3 d in normal mice and 14.4 ± 0.7 d in diabetic mice. As shown in Fig. 1A, B and Additional file 1: Fig. S1A, tumors formed by the colon cancer cell lines (especially HT29 cells and HCT116 cells) in the diabetic mice were notably different from those in the non-diabetic mice (*P* < 0.05), indicating that diabetes is more likely to induce colon tumors in vivo. Therefore, the HT29 cells were used for the following in vitro and in vivo studies.

As the closed links among diabetes, hyperglycemic, and colon cancer, the cellular glucose concentration could be critical in the progression of colon cancer. By assessing cell viability and cell apoptosis assays, we explored the proper concentrations of glucose in HT29 cells for further in vitro analysis. According to two principles: (1), the cell viability was above 80% (Additional file 1: Fig. S1B); (2), the apoptosis rate was below 10% (Additional file 1: Fig. S1C), 2 g·L^−1^ (11.1 mM) and 5 g·L^−1^ (27.8 mM) were selected as the normal glucose and high glucose concentrations for the following experiments, respectively.

We next accessed the in vitro wound healing-based cell migration assay and transwell-based cell invasion assay to test whether LF acts as the inhibitor in a HT29 colon cancer model. As shown in Additional file 1: Fig. S1D and E, LF treatment showed obviously suppressive ability in migration and invasion activities of HT29 cells (comparing with the control, *P* < 0.05), further validating the potential anti-malignancy effect of LF on colon cancer. Taken together, we screened HT29 colon cancer cells as the working model for colon cancer development under a high concentration of glucose (hyperglycemia) and verified the potent function of LF.

### High throughput sequencing identified NT5DC3 as the key factor in the progression of colon cancer under a high concentration of glucose

To evaluate the inner mechanism of the progression of colon cancer under a high concentration of glucose, we performed high throughput RNA sequencing to screen potential candidates that involved in colon carcinogenesis. The GO classification and pathway enrichment of the DEGs were illustrated in Fig. 1C and Additional file 2: Fig. S2A and B. We analyzed the potential cancer-related as well as glucose metabolism-related gene candidates, together with the published information regarding both colon cancer and diabetes in the GEO database [24]. Ultimately, our focus was narrowed to 5'-nucleotidase domain-containing protein 3 (NT5DC3), which was significantly reduced (log2FC = − 1.22) in the HT29 cells. NT5DC3 has been previously reported to be altered in colon cancer [25]. Excitingly, the following epigenetic assessments in the present study further accounted for the dys-expression of NT5DC3 and its key role in the progression of colon cancer under hyperglycemia. DNA methylation microarray showed that LF caused a dramatic alteration of the DNA methylation profiling in the HT29 cells, with 11,038 hyper-methylated genes and 9595 hypo-methylated genes (Additional file 2: Fig. S2C–E). The function enrichment of the differentially methylated genes (DMGs) further represented their potential correlations with T2D (Additional file 2: Fig. S2F). Based on the co-analysis of transcriptomics (Supplementary transcriptomics screening data) and DNA methylation detection (Supplementary DNA methylation profiling data), we found that LF could increase the mRNA expression level of *NT5DC3*, intriguingly, LF could also decrease the DNA methylation level of *NT5DC3* (comparing with the control, *P* < 0.05). Considering the anti-tumor effect of *NT5DC3* gene in tumor models especially in colon cancer has not be clearly uncovered yet, the study on this novel factor might be beneficial to colon cancer pretreatment in clinical area, therefore, we primarily defined *NT5DC3* as the candidate tumor suppressor in inhibiting colon cancer and preliminarily identified the regulation of LF on *NT5DC3* (Additional file 2: Fig. S2C–E). These data indicate that *NT5DC3* is a bona fide downstream target of LF and suggest that *NT5DC3* might be a potential biomarker in colon tumor progression under hyperglycemia.

### NT5DC3 and HKDC1 are regulated by LF

Hexokinase domain component 1 (HKDC1) is reported to play a critical role in the maintenance of glucose homeostasis and there is increasing evidence to suggest that its overexpression may contribute to several types of cancers [26]. To this end, we examined the mRNA and protein levels of NT5DC3 and HKDC1 under normal or high glucose conditions in different types of cells. NT5DC3 expression levels were down-regulated, while HKDC1 expression levels were significantly up-regulated in HT29 cells under a high-glucose treatment (*P* < 0.05), compared to the cells under normal-glucose conditions (Fig. 1D–G). Furthermore, we observed that co-treatment of LF could neutralize the differential expression of NT5DC3 and HKDC1 induced by glucose concentration (comparing with control, *P* < 0.05) (Fig. 1H, I), suggesting the important mechanism underlying LF’s anti-tumor activity via the regulation of NT5DC3 and HKDC1. Moreover, LF-dependent up-regulating of NT5DC3 and down-regulating of HKDC1 expression provided strong evidence that NT5DC3 and HKDC1 might play pivotal roles in the induction of T2D to colon tumor which could be relieved by LF. To exclude the possibility that LF-induced NT5DC3 and HKDC1 expression is caused by indirect effects, we utilized siRNA-mediated inactivation of* NT5DC3* and *HKDC1* in HT29 cells and validated the relationship between the two factors. As shown in Fig. 1J, the level of HKDC1 increased sharply after transfection of *NT5DC3* siRNA under both normal glucose and high glucose (*P* < 0.05), and LF failed to decrease its expression. As expected, after *HKDC1* knockdown, the level of NT5DC3 showed no change in comparison to the control group, while LF up-regulated its expression (Fig. 1K), indicating that LF could suppress the expression of HKDC1 by up-regulating its upstream factor NT5DC3. These data demonstrate that aberrant expression altering of NT5DC3 and HKDC1 induced by intracellular glucose could be directly modulated by LF, underscoring the potential role of LF in colon cancer therapy.

### LF inhibits colon tumor development in T2D mice

**Fig. 2:**
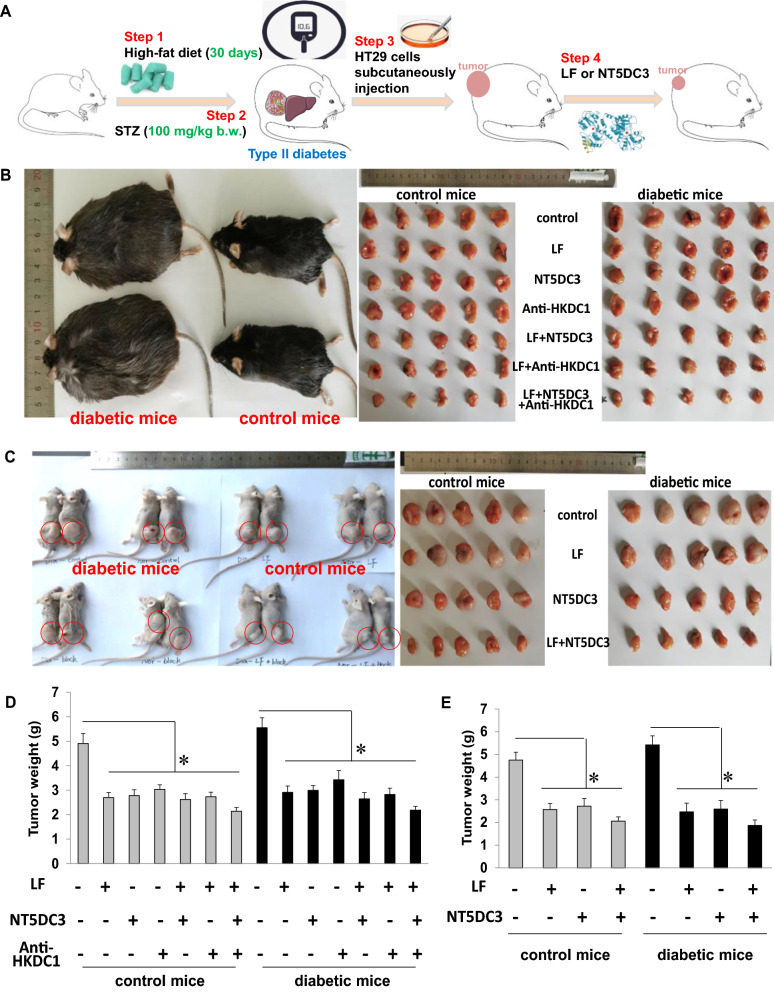
HT29 xenograft tumors in two mouse models. **A** The workflow of HT29 xenograft tumors in mouse models. **B** In the C57BL/6 mouse model, comparison of diabetic mice and normal mice, and tumors dissected from diabetic mice and normal mice implanted with HT29 cells, which were treated with lactoferrin (LF), NT5DC3 protein (NT), HKDC1 antibody (HK), or their combinations. **C** In BALB/c nude mouse model, comparison of diabetic mice and normal mice, and tumors dissected from diabetic mice and normal mice implanted with HT29 cells, which were treated with LF, NT, or their combinations. **D** The average tumor weights of diabetic mice and normal mice in the C57BL/6 mouse model. **E** The average tumor weights of diabetic mice and normal mice in the BALB/c nude mouse model. The tumor group stands for the control. The above data (**D**, **E**) are presented as mean ± SD, *P < 0.05 compared with the control (n = 5)

As the LF-dependent NT5DC3/HKDC1 expression alteration in colon cancer, we sought to determine the relevance of LF and downstream targets NT5DC3/HKDC1 in HT29 tumor model in vivo. To this end, we assessed the HT29 implantation tumor model in immune-competent C57BL/6 mouse by LF, NT5DC3 protein, or HKDC1 antibody or combination treatment both in normal and diabetic mice. The workflow of mice model construction was demonstrated in Fig. 2A. As expected, in the average tumor weight of the C57BL/6 diabetic mice was larger than the corresponding one of C57BL/6 normal mice, the one of control mice was 4.9 ± 0.4 g and the one of diabetic mice was 5.6 ± 0.6 g, indicating that HT29 tumors grow faster under a high glucose environment (Fig. 2B). We observed single LF, NT5DC3 protein, or HKDC1 antibody treatment could partially suppress the tumor growth. However, the tumor growth was significantly inhibited under double combination or triple combination treatment (comparing with the untreated groups, *P* < 0.05) (Fig. 2B, D). To exclude the possibility that the immune system could affect the result of implantation, this assay was also performed in immune-deficient BALB/c nude mice. As is shown in Fig. 2C and E, the tumor average weight of BALB/c control mice was 4.8 ± 0.3 g and the one of diabetic mice was 5.4 ± 0.5 g, indicating that HT29 tumors consistently demonstrated stronger growing ability under a high glucose environment (Fig. 2C). Further, LF and NT5DC3 protein combination significantly inhibited the development of HT29 tumors (comparing with the untreated groups, *P* < 0.05), underscoring the tumor suppressor NT5DC3 corroborates with LF to anti-tumor roles mainly through the activation of the NT5DC3 level in diabetic mouse models (Fig. 2C, E). These results provided evidences that the high risk of diabetic mice in developing colon cancer could be suppressed by LF and NT5DC3.

### LF suppresses 5mC and m^6^A of *NT5DC3*

**Fig. 3:**
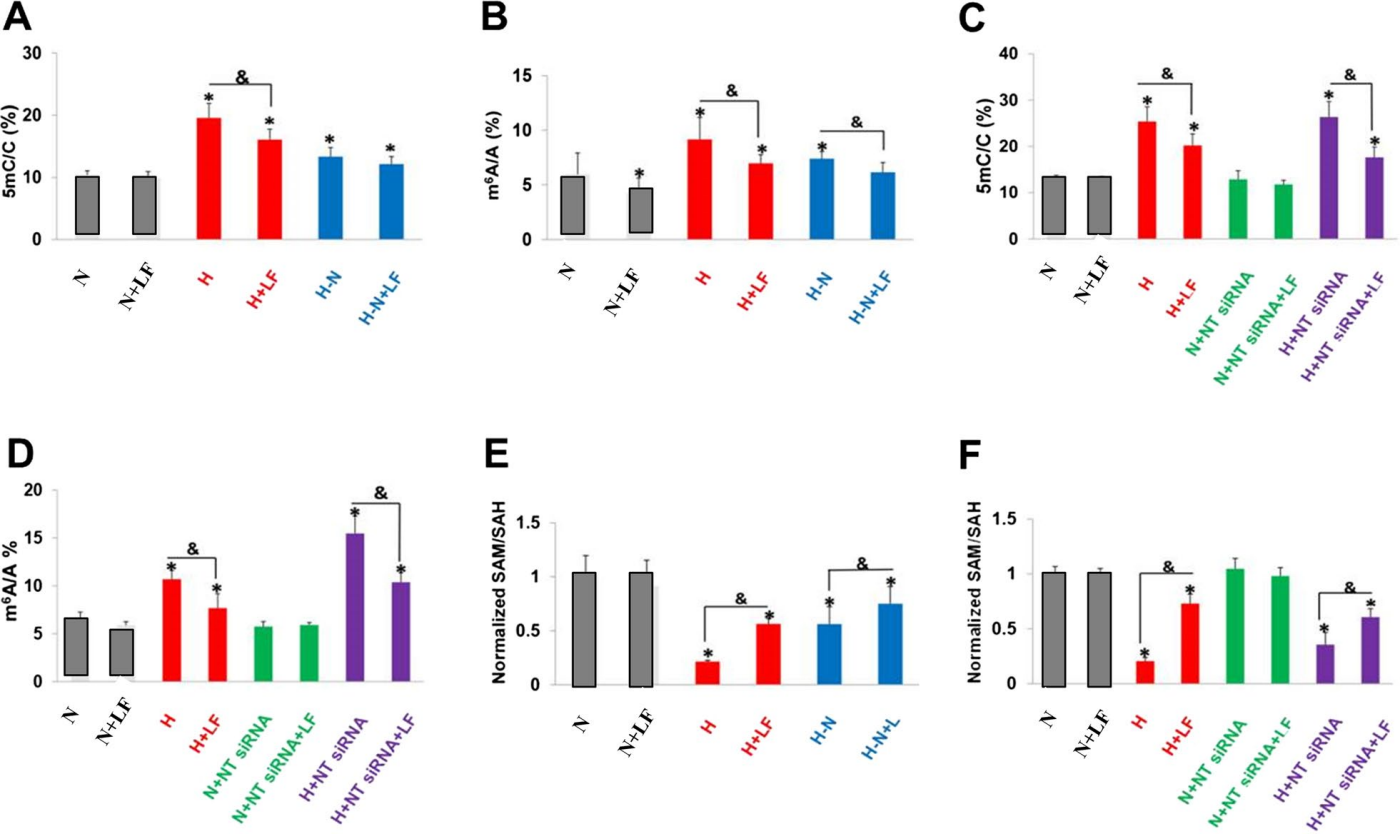
The total 5mC and m^6^A levels, as well as SAM/SAH ratio detected by MS. **A** The levels of 5mC/C under different concentrations of glucose. **B** The levels of m^6^A/A under different concentrations of glucose. **C** The levels of 5mC/C with *NT5DC3* siRNA treatment. **D** The levels of m^6^A/A with *NT5DC3* siRNA treatment. **E** The ratios of SAM/SAH under different concentrations of glucose. **F** The ratios of SAM/SAH with *NT5DC3* siRNA treatment. N stands for normal-glucose (2 g·L^−1^), H stands for high-glucose (5 g·L^−1^), LF stands for lactoferrin, H-N stands for the transfer from high-glucose to normal-glucose. The above data are presented as mean ± SD, *P < 0.05 compared with the normal, & *P* < 0.05 compared with LF treatment group (n = 3)

To elucidate the epigenetic regulation of NT5DC3 in the progression of colon cancer under hyperglycemia condition, DNA 5mC of *NT5DC3* on genome level and RNA m^6^A modification of *NT5DC3* on transcript level were detected. Cells were treated under different concentrations of glucose with or without LF (normal, high, normal + LF, high + LF, high transfer to normal, and high transfer to normal + LF), and the ratio of DNA 5mC/C or RNA m^6^A/A was determined by the liquid chromatography-tandem mass spectrometry/mass spectrometry (LC–MS/MS) technique. As shown in Fig. 3A and B, the ratio of DNA 5mC/C or RNA m^6^A/A was upregulated under a high glucose treatment in comparison to normal glucose conditions (lane 1 vs. 3) and downregulated after the transfer from high to normal glucose conditions (lane 3 vs. 5), and there were significant statistical differences (*P* < 0.05). However, the ratio was not affected by LF treatment under normal conditions (lane 1 vs. 2). Conversely, there was obvious decreasing ratio of DNA 5mC/C or RNA m^6^A/A under a high glucose conditions by LF treatment (lane 3 vs. 4, and lane 5 vs 6, *P* < 0.05), suggesting LF is an unexpected modulator in the global epigenome. Further, we found the knockdown of *NT5DC3* had no obvious effect on the ratio of DNA 5mC/C or RNA m^6^A/A, further indicating NT5DC3 was the downstream of LF-dependent regulation (*P* < 0.05) (Fig. 3C, D).

S-Adenosylmethionine (SAM) is the methyl donor for the biological methylation modifications which regulates the functions of nucleic acids and proteins [2, 27]. Methylation consumes SAM and converts the byproduct, S-adenosylhomocysteine (SAH) [28]. Thus, the methylation index (SAM/SAH ratio) is commonly considered an indicator of cellular methylation potential, if the ratio is lower, the methylation level usually increases [28]. To this end, SAM and SAH concentrations were analyzed to confirm the tight relationship with 5mC and m^6^A, SAM/SAH ratio is negative correlated with the methylation degree of 5mC and m^6^A. The results showed that the SAM/SAH ratio had the same pattern as the ratio of DNA 5mC/C or RNA m^6^A/A shown above, SAM/SAH ratio was sharply lower under high concentration of glucose when compared with the control group (*P* < 0.05), indicating that the methylation degree was upregulated under hyperglycimia, and LF could increase the SAM/SAH ratio significantly, that was to say, LF took effects in downregulating 5mC and m^6^A methylation levels (*P* < 0.05) (Fig. 3E, F). Whereas *NT5DC3* knockdown still had no influence on SAM/SAH ratio (Fig. 3F). These data demonstrate the epigenetic regulation of cellular glucose concentration and LF is mediated by SAM/SAH dependent DNA 5mC or RNA m^6^A proportion, suggesting a high concentration of glucose-induced DNA or RNA methylation could be alleviated by LF treatment.

### The mechanism of the epigenetic regulation of NT5DC3 by LF

**Fig. 4:**
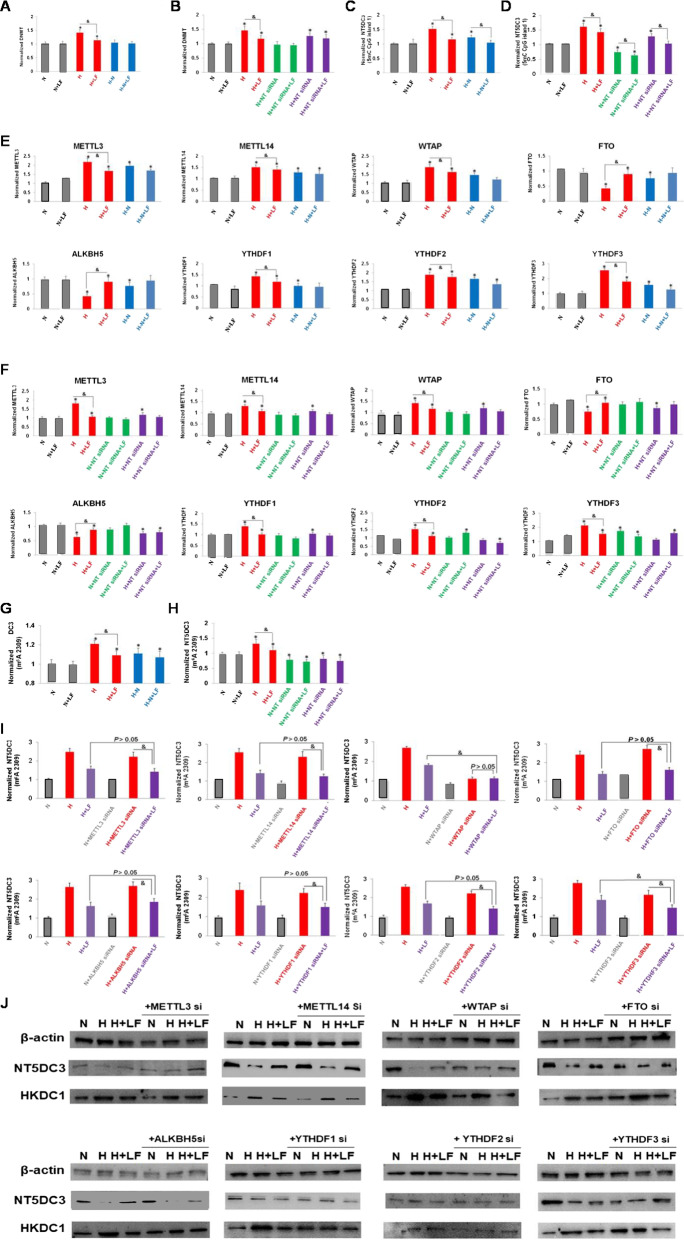
The levels of DNA/RNA methylation-related genes, as well as the normalized *NT5DC3* (5mC CpG island 1/m^6^A 2309 site). **A** The levels of *DNMT* under different concentrations of glucose. **B** The levels of *DNMT* with *NT5DC3* siRNA treatment. **C** The levels of *NT5DC3* (5mC CpG island 1) under different concentrations of glucose. **D** The levels of *NT5DC3* (5mC CpG island 1) with *NT5DC3* siRNA treatment. **E** The levels of eight m^6^A related genes (*METTL3*, *METTL14*, *WTAP*, *FTO*, *ALKBH5*, *YTHDF1*, *YTHDF2* and *YTHDF3*) under different concentrations of glucose. **F** The levels of eight m^6^A- related genes with *NT5DC3* siRNA treatment. This part also verified the results in (**E**). **G** The levels of *NT5DC3* (m^6^A 2309) under different concentrations of glucose. **H** The levels of *NT5DC3* (m^6^A 2309) with *NT5DC3* siRNA treatment. **I** The levels of *NT5DC3* (m^6^A 2309) with the treatment of eight genes siRNA fragments, respectively. **J** The protein levels of NT5DC3 and HKDC1 with the treatment of eight genes siRNA fragments, respectively. N stands for normal-glucose (2 g·L^−1^), H stands for high-glucose (5 g·L^−1^), LF stands for lactoferrin, H-N stands for the transfer from high-glucose to normal-glucose. The above data are presented as mean ± SD, *P < 0.05 compared with the control, & *P* < 0.05 compared with LF treatment group (n = 3).

It is well defined that 5mC DNA methylation modifications are catalyzed by the participation of the *DNMT* [29]. Thus, we firstly detected the expression level of *DNMT* using the same condition, as shown in Fig. 4A and B, *DNMT* level was upregulated under a high glucose treatment which could be inhibited by LF (*P* < 0.05). In conformity with this result, the *NT5DC3* CpG island 1 level was changed at the same pattern (*P* < 0.05) (Fig. 4C, D). On the other hand, the expression levels of RNA methylation-related genes (*METTL3*, *METTL14*, *WTAP*, *FTO*, *ALKBH5*, *YTHDF1*, *YTHDF2* and *YTHDF3*) were also detected by qPCR analysis. All the eight m^6^A-related genes were observed to change significantly under a high glucose in comparison to the normal group, the levels of m^6^A eraser genes (*FTO* and *ALKBH5*) were downregulated, the levels of m^6^A writer genes (*METTL3*, METTL14 and *WTAP*) and m^6^A reader genes (*YTHDF1*, *YTHDF2* and *YTHDF3*) were all upregulated under the high glucose conditions (*P* < 0.05), indicating that m^6^A was activated under hyperglycemia (Fig. 4E, F). However, LF could upregulate the levels of m^6^A eraser genes and downregulate the levels of m^6^A writer genes and reader genes under the high glucose conditions (*P* < 0.05), but not under normal glucose conditions, further verifying that LF selectively decreased the degree of m^6^A under hyperglycemia (Fig. 4E, F). Furthermore, the RNA m^6^A of *NT5DC3* level was obviously upregulated under a high glucose in comparison to the normal group, and LF significantly attenuated the *NT5DC3* m^6^A level at site 2309 (*P* < 0.05) (Fig. 4G, H). These results demonstrate LF could affect the 5mC and m^6^A machine genes’ expression to control the methylation status of *NT5DC3*.

Next, to determine which gene was specifically responsible for the *NT5DC3* m^6^A level modulating at site 2309, we performed an m^6^A related siRNA knockdown screen to identify the NT5DC3 regulator: (1) MS analysis to test the *NT5DC3* 2309 m^6^A level; (2) Western blot assay to test NT5DC3 protein level. Interestingly, the knockdown of *WTAP* could abrogate the changing of the *NT5DC3* m^6^A or NT5DC3 protein levels after LF treatment under a high glucose conditions, and *ALKBH5* siRNA group (panel 5), *YTHDF*1 siRNA group (panel 6) and *YTHDF3* siRNA group (panel 8) showed the similar pattern of changes as *WTAP* siRNA group indicating that these m^6^A-related genes could be regulated by LF under hyperglycemia, and *WTAP* showed the stongest changes (*P* < 0.05) (Fig. 4I, J). The data suggested that these genes especially *WTAP* might be required for LF dependent m^6^A (2309 site) of *NT5DC3* and subsequently the protein expression of NT5DC3. As for HKDC1 protein, we found that its expression level was reversely regulated in comparison to NT5DC3, in both the *WTAP* siRNA group and LF treatment group (*P* < 0.05) (Fig. 4J), further proving that LF could suppress the progression of colon cancer under a high concentration of glucose through regulating the *WTAP*/m^6^A/ *NT5DC3*/*HKDC1* axis.

To exclude the possibility that this regulation mechanism was cell line-specific, we performed the same experiment in HCT116 cell line, which had the same growth ability as HT29 cells in diabetic induced tumors. As was shown in Additional file 3: Fig. S3A–C, the ratio of DNA 5mC, RNA m^6^A, and SAM/SAH increased significantly under a high glucose condition in comparison with the normal conditions which were suppressed by LF treatment but not *NT5DC3* knockdown (*P* < 0.05). Further, we found that *DNMT* or *WTAP* were consistently required for DNA 5mC or RNA m^6^A regulation, respectively (Additional file 3: Fig. S3D–I). These data indicate that epigenetic regulation of *NT5DC3* by LF through *DNMT*-mediated DNA 5mC or *WTAP*-mediated RNA m^6^A has broad relevance in colon cancer cells.

### The epigenetic regulation of NT5DC3 and potential consequences in vivo

**Fig. 5:**
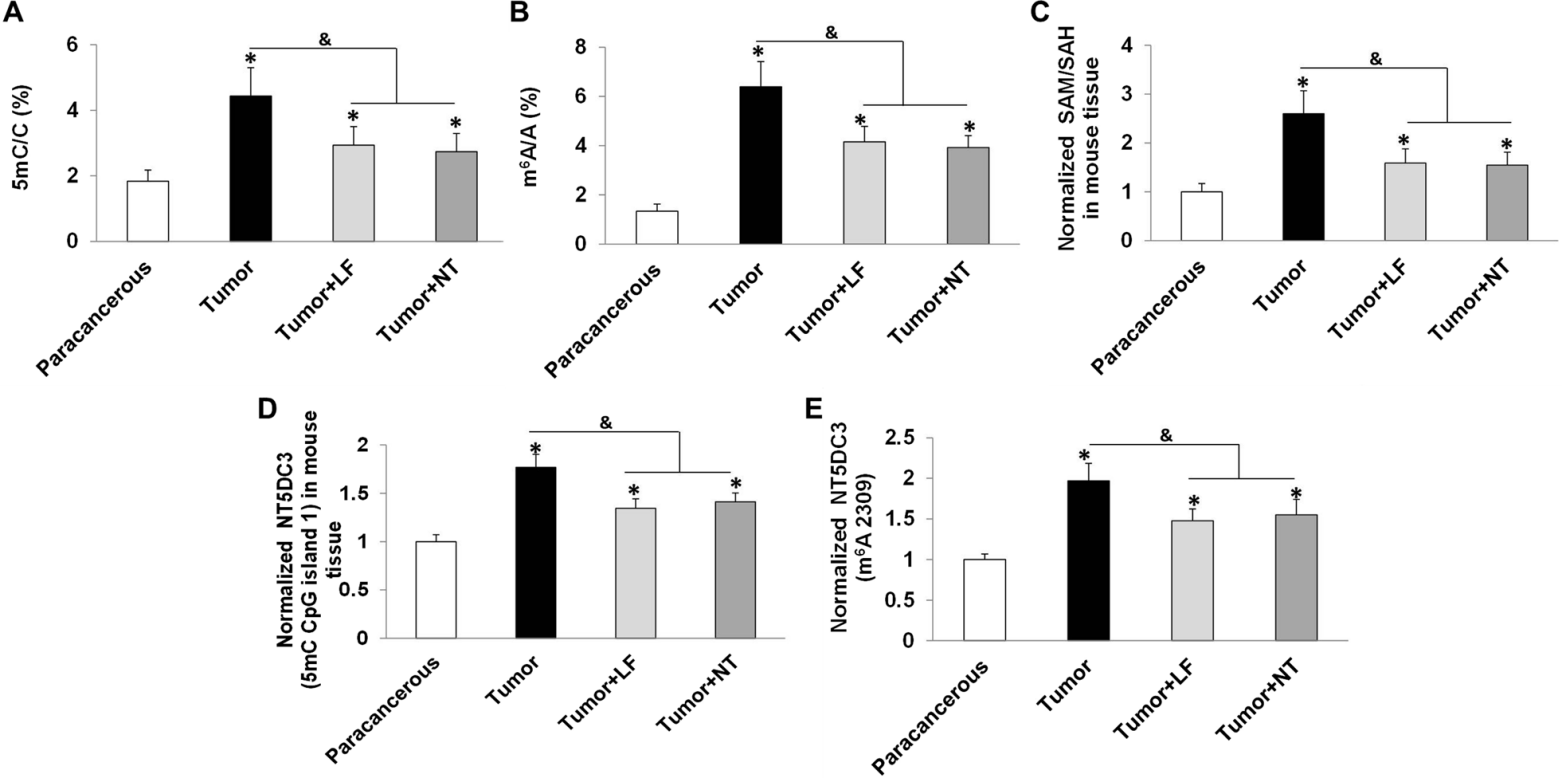
Mythelation detection of *NT5DC3*. **A** The 5mC/C in C57BL/6 mice paracancerous tissue and tumor tissue samples by MS. **B** The m^6^A/A in C57BL/6 mice paracancerous tissue and tumor tissue samples by MS. **C** The ratio of SAM/SAH in C57BL/6 mice paracancerous tissue and tumor tissue samples by MS. **D** The levels of *NT5DC3* (5mC CpG island 1) in C57BL/6 mice paracancerous tissue and tumor tissue samples by q-PCR. **E** The levels of *NT5DC3* (m^6^A 2309) in C57BL/6 mice paracancerous tissue and tumor tissue samples by q-PCR. Tumor stands for the control group, LF stands for lactoferrin group. The above data are presented as mean ± SD, *P < 0.05 compared with the normal, and & *P* < 0.05 compared with tumor group (n = 3)

We further investigated the epigenetic regulation of *NT5DC3 *in vivo. To this end, the ratios of 5mC/C, m^6^A/A, or SAM/SAH at the tumor tissue or normal precancerous tissue in the C57BL/6 mice were measured. As expected, in comparison to normal precancerous tissues, all these tested ratios increased in tumor tissues, whereas LF significantly downregulated them (*P* < 0.05), indicating that the ratios of 5mC/C, m^6^A/A, SAM/SAH were consistently controlled by glucose and LF in vivo (Fig. 5A–C). Moreover, 5mC (*NT5DC3* CpG island 1) and m^6^A (*NT5DC3* 2309 site) in mice tumor tissues were higher than the ones in precancerous tissues but downregulated by LF treatment as well (*P* < 0.05). These data demonstrate that regulation of NT5DC3 by 5mC and m^6^A is conserved from mice to humans, underlying the importance of this instinct regulation mechanism in vivo.

### NT5DC3 expression is the biomarker for T2D or T2D/colon cancer coexistence patients

**Fig. 6:**
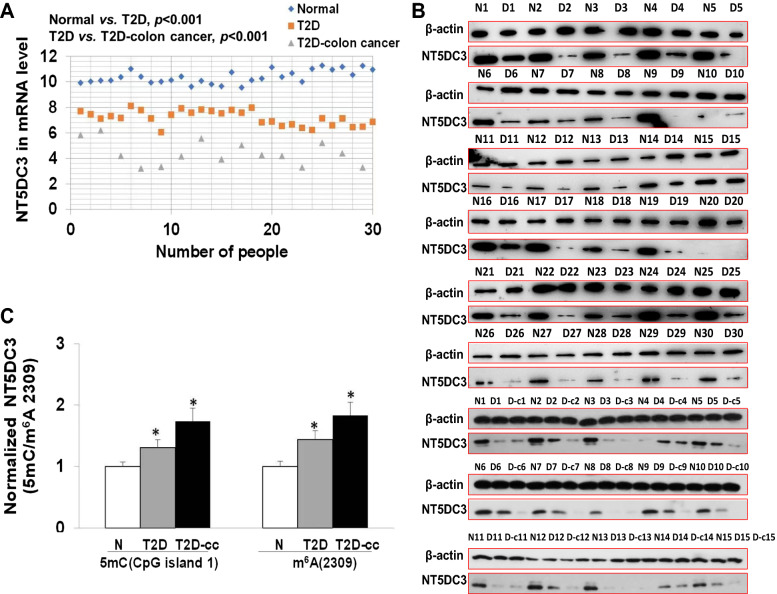
Detection of NT5DC3 in human blood samples. **A** The mRNA level of *NT5DC3* by q-PCR. **B** The protein level of NT5DC3 by western blotting. **C** The 5mC (CpG island 1) and m^6^A (2309 site) of *NT5DC3* in blood samples. N1-30 stands for the healthy volunteer group, T2D1-30 and D1-30 stand for the T2D patient group. T2D-cc1-15 and T2D-c1-15 stand for the T2D-induced colon cancer patient group. The above data are presented as mean ± SD, *P < 0.05 compared with the healthy volunteer group (n = 75)

Because the above data indicate that *NT5DC3* regulation plays a critical role in colon cancer development in T2D mice in response to LF, it is very likely that NT5DC3 could be the biomarker for T2D-induced colon cancer. To corroborate this finding, we collected 75 clinical blood samples from healthy people (n = 30), T2D patients (n = 30), and T2D/colon cancer coexistence patients (n = 15). Then, we tested the mRNA and protein levels of NT5DC3 and found a decrease of NT5DC3 in T2D patients in comparison to the healthy people group, further confirming that the expression levels of *NT5DC3* reflect the blood glucose concentration (Fig. 6A, B). Notably, the NT5DC3 expression showed a dramatic reduction in the clinical T2D/colon cancer coexistence group which was consistent with our above finding (*P* < 0.05). Moreover, the *NT5DC3* 5mC at CpG island 1 or *NT5DC3* m^6^A 2309 site methylation levels among these three groups were increased in gradient (*P* < 0.05), further indicating the aberrant epigenetic profiling occurs during T2D or colon cancer progression in T2D patients (Fig. 6C). These results suggest that the NT5DC3 mRNA and protein levels are a bona fide biomarker in distinguishing healthy volunteers and T2D patients, and even T2D/colon cancer coexistence patients. These results also demonstrate the potentness of monitoring NT5DC3 in the prognosis of T2D or T2D-induced colon cancer. In the clinical field, whether NT5DC3 could be a marker in diagnosing T2D patients who have a higher risk of getting colon cancer required more patient surveys and sample verification.

## Discussion

*NT5DC3*, a member of the 5'-nucleotidase domain-containing family, codes for a largely uncharacterized transmembrane protein [30]. The aberrant expression of 5ʹ-nucleotidase domain-containing family members is reportedly associated with abnormalities, cancer, endocrine system disorders, and organismal injury [30]. In this study, we found NT5DC3 as a key target of diabetes and colon cancer under hyperglycemia. Our identification and development of colon cancer cells (HT29 and HCT116 cell lines) induced mouse tumor models provide a platform to investigate the effect of natural dietary component LF. On the other hand, based on the co-analysis of transcriptomics and DNA methylation detection, we defined *NT5DC3* as the tumor suppressor in inhibiting colon cancer and preliminarily identified the regulation of LF on *NT5DC3*.

Several studies have demonstrated that LF can be applied as a candidate treatment for diabetes or to efficiently inhibit the growth of tumors and reduce susceptibility to cancer [4, 19–23]. Notably, the methylation of the promotor and the first intronic region of LF, associated with an increased incidence of cancer, has been observed in cancers of the breast, lung, prostate, etc. [31–33]. Alterations of the methylation profile have been described in T2D [8], however, information regarding the relationship between LF, methylation, and T2D remains limited. In the present study, LF was found to decrease 5mC and m^6^A methylation of *NT5DC3* in colon tumor cells and xenografted colon tumors implanted in T2D mice, thereby suggesting its potential to suppress the progression of colon cancer under a high concentration of glucose (hyperglycemia).

By using epigenetic and biochemistry techniques, we successfully discovered and verified the modification sites of *NT5DC3* regulated by LF. LF was proved to destroy the bridge between T2D and colon cancer through the suppression of epigenetic modification levels of 5mC (CpG island 1) and m^6^A (2309) of *NT5DC3*. We also found that *WTAP* played a key role in m^6^A (2309) of *NT5DC3*, which subsequently affect the expression levels of *NT5DC3*/*HKDC1*. *WTAP*-mediated m^6^A methylation has a crucial role in tumorigenesis. WTAP acts as a sensitive marker in gastrointestinal cancer [34], non-small cell lung cancer [35], breast cancer [36], etc. It is reported that WTAP involves in hepatocellular carcinoma (HCC) oncogenesis via regulation of the HuR-ETS1-p21/p27 axis [37]. Overexpression of *WTAP* facilitates renal cell carcinoma by stabilizing CDK2 transcript [38], and *WTAP* promotes the invasiveness of glioblastoma through enhancing the activity of EGFR [39]. Moreover, *WTAP* cooperates with *METTL3* and *METTL14* to promote cell cycle transition in the MCE of adipocyte differentiation [40]. Those findings highlight *WTAP* as a potential therapeutic target for cancer treatment, however, little has been known about the molecular function of *WTAP* in the progression of colon tumors under a high concentration of glucose. Our study uncovered that *WTAP* participated in m^6^A (2309) of *NT5DC3* to suppress NT5DC3 expression thus, LF-dependent *WTAP* expression inhibition exhibited a novel pathway to suppress the progression of colon tumor under a high concentration of glucose. However, how LF inhibits WTAP expression needs further elucidation. These discoveries about the mechanism of *NT5DC3* in suppressing colon cancer progression under hyperglycemia, combined with the positive regulation of LF on *NT5DC3*, will expand current knowledge of the functions of bioactive proteins within different research layers. Moreover, the results from clinical blood samples defined *NT5DC3* as a sensitive biomarker to distinguish T2D patients from healthy volunteers, and the expression levels of *NT5DC3* in the patients who had the coexistence of colon cancer and T2D were continuously lower than the ones in T2D patients. Further investigation should be conducted to ascertain whether this mechanism holds true in more clinical T2D-induced colon tumor patients, or whether LF inhibits the progression of the two diseases via acting on *WTAP*/m^6^A/*NT5DC3*/*HKDC1* axis in the clinical area. Based on a large number of toxicological experiments, LF is regarded as GRAS (generally recognized as safe) by FDA, thus, the dosage of 250 mg·kg^−1^ body weight (b.w.) (as 3.1 μM·kg^−1^ b.w.) used in the present study might be completely accepted in future clinical treatments, especially in the prevention from T2D to colon cancer, which also deserves our further investigation.

In brief, we revealed that lactoferrin acts as a suppressor in the progression of colon cancer under a high concentration of glucose, by which epigenetically regulating glucose-dependent *DNMT*/5mC or *WTAP*/m^6^A/*NT5DC3*/*HKDC1* axis. Lactoferrin could regulate *WTAP* and inhibit m^6^A of *NT5DC3* at 2309 site, and subsequently affect the expression levels of NT5DC3/HKDC1 proteins in vitro and in vivo. Our study reveals that lactoferrin and its downstream target *NT5DC3* represent a new light in clinical T2D-induced colon cancer prognosis and treatment, underscoring the potential application of natural dietary mediated tumor suppression. If possible, the measurement of NT5DC3 expression level might be developed as a sensitive biomarker in clinical prediction models, to distinguish whether T2D patients are susceptible to developing colon cancer.

### CRediT authorship contribution statements

HYL, HPJ, XCZ, BYZ– Planning and execution of research work. HPJ and XCZ– Experimental design and statistical analysis of research work. HYL and BYZ–Supervision and interpretation. All authors read and approved the final manuscript.

### Ethics declaration

The animal experiments were approved by the Ethics Committee of Chinese Academy of Agriculture Sciences (Beijing, China; Permission No. IAS2020-90). All patients submitted the informed written consent to utilize biological specimens for investigational procedures, according to the Ethics Committee approval of Beijing Friendship Hospital, Capital Medical University (Permission No. 2020-P2-175-02, as Supplementary Ethics Committee approval in Data and Code Availability section).

## Materials and methods

### Cell culture and viability detection

GES1 cells, MGC803 cells, HK2 cells, SW839 cell, NCM460 cells, HT29 cells, HCT116 cells, and SW620 cells were cultured in RPMI1640 medium (containing 2 g·L^−1^ (11.1 mM) glucose) with 10% FBS, at 37 °C in 5% CO_2_ and 95% saturated atmospheric humidity. The cells were plated into 96-well plates (1 × 10^4^ cells/100 μL medium) and incubated for 24 h, then the medium was replaced with 100 μL fresh medium containing different concentrations of glucose (0, 1, 2, 3, 4, 5, 6, 7 and 8 g·L^−1^, also as 0, 5.6, 11.1, 16.7, 22.2, 27.8, 33.3, 38.9 and 44.4 mM, respectively). Then the cells were cultured for another 48 h, and the CCK-8 kit was applied to measure cell viability. The absorbance at 490 nm was determined by a Microplate Reader (Thermo Fisher Scientific). The cell viability = (Value test − Value blank) / (Value control − Value blank) × 100%. The dosages with the viabilities greater than 80% meanwhile significantly different from the control (*P* < 0.05) were chosen as the appropriate concentrations of glucose used in the following experiments.

### Cell apoptosis assay

Cells grew in 6-well plates treated with a growth medium containing increasing concentrations of glucose (1, 2, 3, 4, 5, and 6 g·L^−1^, respectively). The cells were collected and resuspended in 250 μL binding buffer, then treated with 15 μL FITC-Annexin V buffer and 30 μL propidium iodide (PI) buffer (10 g·L^−1^, as 15.0 mM), gently vortexed and incubated for 15 min in the dark at 25 °C. Subsequently, 300 μL binding buffer was added into each tube and then analyzed by flow cytometry (BD) within 1 h.

### Preparation of lactoferrin with approximate 50% iron saturation

Lactoferrin with 100% iron saturation (Holo-LF) was purchased from Sigma (USA). 11 g Holo-LF was added with 300 mL citrate buffer (pH = 5), and adjusted PH value to 8.2, to promise iron saturation as 49%-51% according to the fitting equation (Y = 0.0643*2.27045^X^, Y stands for LF iron saturation, X stands for PH value). Then the solution was concentrated and desalted through a 30 kDa ultrafiltration membrane. When the conductivity was less than 1.5 ms·cm^−1^, the volume was concentrated 5 times and lyophilized (Chinese Invention patent application, Application publication number: CN 110655570 A).

### Cell invasion and migration analyses

In the migration test, the upper chambers were seeded with 5 × 10^3^ cells in 200 μL serum-free medium containing normal or high glucose, respectively, and 400 μL of medium (2 g·L^−1^ or 5 g·L^−1^ glucose, accordingly) with 15% FBS was added to the lower chambers. Lactoferrin with approximate 50% iron saturation (LF, 0.5 g·L^−1^, as 6.3 μM) prepared above was added to the upper chamber and cocultured for 24 h, then cell invasion was detected by calculating three random captured pictures [20].

In the invasion test, HT29 cells were plated in a 6-well plate and incubated for 24 h to achieve a cell density greater than 90%. A single lesion with a width of approximately 5.0 mm was scratched across the cell monolayer by mechanical scraping. The cells were then incubated with LF (0.5 g·L^−1^) dissolved in a normal (2 g·L^−1^) or high glucose (5 g·L^−1^) medium. The width of the scratch wound was photographed and scanned again 24 h later, and the recovery rate was measured [20].

### mRNA sequencing and DNA methylation profiling

Total RNA (1 μg) extracted from each cell sample was firstly utilized to poly-d(A)-RNA isolation with NEBNext Magnetic Oligo d(T)25 Beads (NEB), and then used for mRNA library preparation with an RNA Library Prep Kit for Illumina (NEB) according to the instruction procedures. All of the libraries were subjected to 150 bp pair-end sequencing on an Illumina HiSeq2000 platform. After sequencing, trimmed and cleansed reads were analyzed by using the Bowtie2 suite to align to the hg19 reference genome and count normalized transcript abundance. Differentially expressed genes (DEGs) were calculated using the DESeq2 package and further analyzed based on GO biological processes, molecular functions, and the KEGG pathway. DNA methylation profiling was studied using the Infinium Human Methylation EPIC Bead Chip. In brief, 6 samples of 500 ng genomic DNA isolated from each treatment (HT29 cells as control group, HT29 cells treated with 0.5 g·L^−1^ LF in high glucose medium as the LF group, 4 biological repetitions) were treated with EZ DNA methylation kit (Zymo Research), the targets were prepared, labeled and hybridized with the kits and reagents indicated by the Infinium HD Methylation Assay Protocol Guide (15019519B). Processed methylation chip after single base extension and staining was scanned using an iScan reader (Illumina). Generated microarray data were analyzed using Genome Studio software v2011.1 (Illumina), and for quality control, methylation measures with a detection p-value > 0.05 and samples with a CpG coverage < 95% were removed. After initial normalization using internal controls in the Genome Studio software, the methylation levels of CpG sites were calculated as β-values (β = Intensity (methylated)/intensity (methylated + unmethylated)). The data were further normalized and the differential DNA methylation was assessed using the IMA 3.12-R package. *P*-values were calculated by t-test corrected for multiple hypotheses testing by the Benjamini–Hochberg method in combination with the Illumina custom false discovery rate (FDR) model (www.tandfonline.com). A threshold for differential DNA methylation was set at FDR-corrected *P*-value lower than 0.05. The functions of the associated genes were further studied based on GO and KEGG pathway analysis using KOBAS.

### Animal models

The animal experiments were approved by the Ethics Committee of Chinese Academy of Agriculture Sciences (Beijing, China; permission number: IAS2020-90), conforming to internationally accepted principles in the care and use of experimental animals (NRC, 2011). All surgical procedures were performed under sodium pentobarbital anesthesia, and all efforts were made to minimize the suffering of the mice.

In C57BL/6 mouse model, 80 male mice (18–22 g) were randomly divided into two parts (16 groups in total): groups in the first part were normal control without any treatment, tumor control treated with HT29 cells implantation, tumor control + LF, tumor control + NT5DC3 protein (Novus Biologicals, USA), tumor control + HKDC1 antibody (Abcam, USA), tumor control + LF + NT5DC3 protein, tumor control + LF + HKDC1 antibody, tumor control + LF + NT5DC3 protein + HKDC1 antibody; while groups in the second part included diabetic mice, diabetic mice implanted with HT29 tumor (dia-tumor mice), dia-tumor mice + LF, dia-tumor mice + NT5DC3 protein, dia-tumor mice + HKDC1 antibody, dia-tumor mice + LF + NT5DC3 protein, dia-tumor mice + LF + HKDC1 antibody, dia-tumor mice + LF + NT5DC3 protein + HKDC1 antibody. The 40 diabetic mice were fed with a high-fat diet for 30 days continuously, and the mice were intraperitoneally administered with streptozocin (STZ, 100 mg·kg^−1^ (0.38 mM·kg^−1^) b.w.) once on the 31st day. Then the indicators including fasting blood glucose detection (FBG), oral glucose tolerance test (OGTT), glycated serum protein (GSP) and serum insulin (INS) of mice were detected on the 33rd day to confirm the successful model construction (data in Additional file 7: Table S4). 1 × 10^8^ HT29 cells in 150 μL matrigel medium (BD) were subcutaneously injected into the back of each mouse, and when the tumors volume reached 100–120 mm^3^, the total 80 mice were treated with LF (250 mg·kg^−1^ b.w.), NT5DC3 protein (50 mg·kg^−1^ (0.94 mM·kg^−1^) b.w.), HKDC1 antibody (50 mg·kg^−1^ (0.42 mM·kg^−1^) b.w.), or their combinations, respectively. LF was orally administered by gavage, while NT5DC3 protein or HKDC1 antibody was injected through the tail vein every two days at the same time. All the mice were sacrificed on the 28th day, and the tumors were weighed.

In BALB/c nude mouse model, 50 male BALB/c nude mice (18–22 g) were randomly divided into two parts (10 groups in total): groups in the first part were normal control without any treatment, tumor control treated with HT29 cells implantation, tumor control + LF, tumor control + NT5DC3 protein, tumor control + LF + NT5DC3 protein; while groups in the second part included diabetic mice, diabetic mice implanted with HT29 tumor (dia-tumor mice), dia-tumor mice + LF, dia-tumor mice + NT5DC3 protein, dia-tumor mice + LF + NT5DC3 protein. The diabetic mice model was constructed and protein treatments were performed as described in the C57BL/6 mouse model. All the mice were sacrificed on the 28th day, and the tumors were weighed.

In the two mouse models, tumor diameters were detected with a caliper every 4 days, and tumor volume was calculated using the following formula: tumor volume (mm^3^) = 0.5 × length (mm) × width (mm)^2^. Individual tumor suppression rate (%) = (the average tumor weight in the control group − the individual tumor weight in the LF treatment groups) / the average tumor weight in the control group × 100%, as the average tumor weight in the control group was calculated by each tumor weight in the control group. Relative tumor volume (RTV, %) = detected volume / volume before dosing × 100% [20].

### The 5mC detection and m^6^A detection

According to the method [41], 1 µg DNA was denatured at 100 °C for 5 min and subsequently chilled at 4 °C for 10 min. One-tenth volume of 0.15 M ammonium acetate (pH 7.5) and 2.5 units of DNase I (TransGen) were added, then the mixture was incubated at 37 °C for 4 h. 2 units of Alkaline Phosphatase (TaKaRa) was added into the solution and incubated for an additional 3 h at 37 °C. Thereafter, the mixture was incubated for 12 h at 37 °C with 40 units of Exonuclease I (TaKaRa). The complete lysis mixture was placed in a refrigerator at 4 °C for LC–MS/MS detection [42]. 1 μg genomic DNA was hydrolyzed by utilizing a DNA degrease Plus™ kit following the manufacturer’s instructions and according to the previous method [43]. In both test samples and standards, the hydrolyzed DNA was analyzed by liquid chromatography-electrospray ionization tandem mass spectrometry with multiple reaction monitoring (LC–ESI–MS/MS-MRM), and the MRM method was applied to monitor three transitions for each analysis, the experiment parameters in the 5mC/m^6^A detection by MS were demonstrated in Additional file 4: Table S1. Finally, the total amount of 5mC in test samples from the 5mC MRM peak area was calculated by dividing the sum of the 5mC and cytosine peak areas (5mC/C) [43].

Purified total mRNA (200 ng) was digested to its constituent mono-nucleosides according to previous method [42]. MRM mode was applied for the UPLC-MS/MS analysis through monitoring transition pairs in the Additional file 4: Table S1.

### Detection of 5mC- and m^6^A-related genes

100 ng total RNA from cells samples or blood samples was extracted, the total RNA samples were transcribed into cDNA (42 °C for 10 min, 65 °C for 10 s, stored at 4 °C) by PrimeScript™ RT reagent Kit (TaKaRa). Primers of evaluated genes including *NT5DC3*, *HKDC1*, Homo sapiens DNA methyltransferase 1 (*DNMT*), methyltransferase-like 3 (*METTL3*), methyltransferase-like 14 (*METTL14*), Wilms' tumor 1-associated protein (*WTAP*), fat mass, and obesity-associated factor (*FTO*), AlkB homologue 5 (*ALKBH5*), YTH N6-methyladenosine RNA binding protein 1 (*YTHDF1*), YTH N6-methyladenosine RNA binding protein 2 (*YTHDF2*), YTH N6-methyladenosine RNA binding protein 3 (*YTHDF3*) and *GAPDH*, as well as siRNA fragments of these genes, were outlined in Additional file 5: Table S2, and *GAPDH* was utilized as the internal reference to assure the equal loadings. qRT-PCR was performed using 96-well microwell plates in a total volume of 20 μL, containing 1 μL template cDNA (10 ng·μL^−1^), 0.5 μL forward primer (10 μM), 0.5 μL reverse primer (10 μM), 10 μL of TB Green® Fast qPCR Mix (TaKaRa). The q-PCR reactions were performed at 95 ℃ for 3 min, followed by 40 cycles of 95 ℃ for 10 s, 60 ℃ for 30 s by using two-step qRT-PCR. All qRT-PCR reactions were performed.

### Methylation sites verified by SELECT qPCR

DNA methylation region of *NT5DC3* (5mC, CpG island 1) determination and primer design principles: DNA sequence of *NT5DC3* (GenBank Reference Sequence: NM_001031701.3) was harvested using NCBI website and MethPrimer (http://www.urogene.org/cgi-bin/methprimer/methprimer.cgi) was utilized for CpG island prediction (CpG island 1: 47–295 bp) and primer design. When DNA is subjected to bisulfite conversion, the bisulfite-sensitive unmodified cytosines (C) are converted to uracils (U) and further replaced by thymidines (T) in PCR amplification, while the methylated cytosines (5mC) could survive the bisulfite conversion and remain unchanged. From the primer candidates provided by MethPrimer, one pair with a high GC content and an annealing temperature close to 60 °C was chosen as the general primers for assessing the methylation status of the predicted CpG island 1 of *NT5DC3* (Additional file 5: Table S2).

Protocols of DNA methylation site (5mC CpG island 1) detection: Genomic DNA (gDNA) was extracted using a DNeasy Blood & Tissue Kit (QIAGEN). 1 μg gDNA of each sample, respectively, was subjected to bisulfite conversion using a DNA bisulfite conversion kit (QIAGEN) following the manufacturers' instructions. A thermocycler was used for the conversion with the following procedure: 95 °C for 10 min and 64 °C for 60 min. After bisulfite conversion, gDNA was purified and used for PCR analysis. The specific primers used were listed in Additional file 5: Table S2. The methylation rate was calculated using the ΔΔCt method: methylation rate (%) = 100%/2^ΔΔCt^.

RNA methylation site of *NT5DC3* (m^6^A, 2309) determination and primer design principles: DNA sequence of *NT5DC3* (GenBank Reference Sequence: NM_001031701.3) was firstly harvested using the NCBI website, and three kinds of sequence that could undergo m^6^A methylation, namely GGACU(T), GAACU(T) and GAACA, were screened. Furthermore, the m^6^AVar database (http://m6avar.renlab.org/index.html) was utilized for the m^6^A site prediction, and chr12:101471023( +), namely m^6^A 2309, was finally chosen for the following investigation (labeled as site X). The nearest adenine (A) on the 5’ upstream of and at least six bases away from the site X was labeled as site N. The RNA methylation-specific primers for both site X and N were designed, respectively. Site X was regarded methylated if the Ct value in site X PCR detection was larger than that in site N detection.

### Protocols of RNA methylation site (m^6^A 2309) detection

*NT5DC3*-2309 methylation in RNA level was detected mainly through three steps by SELECT qPCR, which were conducted by following Xiao’s protocol and previous references [42, 44, 45]. Firstly, the total RNA (1500 ng) was mixed with 40 nM up primers (Additional file 5: Table S2), 40 nM down primers (Additional file 5: Table S2), and 5 μM dNTP (NEB) in 1.7 μL 10 × CutSmart buffer (NEB), 20 μL total volume. Then the RNA and primers were incubated as the reference [46] introduced, then 20 μL qPCR reaction system was set up and contained 5 μL of the final reaction mixture, 200 nM SELECT primers (see *NT5DC3* select in Additional file 5: Table S2), and TB Green® Fast qPCR Mix (TaKaRa). SELECT qPCR was performed with the following program: 95 °C for 5 min; 95 °C for 10 s, 60 °C for 35 s, 40 cycles in total; 95 °C for 15 s; 60 °C for 1 min; 95 °C for 15 s; then hold at 4 °C. Ct values of samples were normalized to their corresponding Ct values of *GAPDH*.

### SAM, SAH detection by MS

SAM and SAH were quantified by LC–MS/MS as described previously [27, 47], with minor modifications to run on UPLC coupled to a XEVO TQ-S micro mass spectrometer. Cell samples were washed three times with ice PBS, followed by bead-beating in 80% methanol: water (LC–MS grade methanol, Thermo Fisher Scientific) at − 20 °C. The extraction mixture was verting for 10 s, and then ultrasonication for 30 min, and centrifuged for 15 min at 12,000 *g*·min^−1^. The supernatants were transferred to an autosampler vial, and 5 µL of the mixture was then used for UPLC-MS/MS analysis (Waters). The separation was performed on a BEH Amide column (130 Å, 1.7 µm, 1 mm × 100 mm, 1/pkg, Waters, USA) for nucleosides. Mobile phases consisted of: (A) 100% water, containing 0.1% formic acid, and (B) 100% acetonitrile containing 0.1% formic acid. The following gradient and mass spectrometer operation were applied as reference described [48, 49]. The precursor → product transitions for SAM (*m/z* 399.3 → 250.3) and SAH (*m/z* 385.3 → 136.3) were monitored.

### Western Blotting analysis

HT29 or NCM460 cells were lysed by RIPA buffer, and the total protein concentration was measured using a BCA kit (Beyotime). The antibodies included: anti-human NT5DC3 (PA5-70919, Invitrogen), anti-human HKDC1(PA5-35894, Invitrogen), and anti-human β-actin (PAB0865, Enzo Life Sciences). For western blotting, the primary antibodies were diluted at 1:1000; the second antibodies were used at 1:3000 dilution. The signals were captured and analyzed by Clinx ChemiCapture software (Clinx).

### Clinical samples collection

From July 2020 to October 2020, 30 patients with T2D and 15 T2D/colon cancer coexsitence patients (colon cancer was diagnosed two years after the T2D diagnosis) were enrolled in the present study. Patients' characteristics are outlined in Additional file 6: Table S3. All patients submitted the informed written consent to utilize biological specimens for investigational procedures, according to the Ethics Committee approval of Beijing Friendship Hospital, Capital Medical University (Permission No. 2020-P2-175–02, as Supplementary Ethics Committee approval in Data and Code Availability section). Blood samples of the patients were taken upon admission on the morning, and the patients did not take any food or water 12 h strictly before the blood was sampled. As the normal control group, 30 healthy people were selected and enrolled in the study. Their blood samples were collected upon admission in the morning. The blood samples of each patient were divided into two parts, 4 mL was collected into RNAase-free tubes (BD) containing preservation solution for DNA/RNA isolation, 2 mL was collected into aseptic anticoagulant tubes (BD) for protein detection.

400 μL lysis buffer was added into each 200 μL blood sample and vortexed for 30 s, which was centrifuged (10,000 rpm, 1 min) later, and the supernatant was removed. The nuclear precipitation was treated with several types of buffer and the total genome DNA was extracted, and the DNA level of *NT5DC3* was measured by qPCR, as mentioned above. DNA methylation (CpG island 1) and RNA methylation (2309 site) of *NT5DC3* were measured by SELECT-qPCR, according to the protocol described above.

100 μL RIPA lysis buffer (with protease inhibitors) was added into each 10 μL blood sample and vortexed for 5 min, then 30 μL 5 × loading buffer was added and boiled for 15 min, and then the protein level of NT5DC3 was detected by western blotting.

### Statistical analysis

All data were presented as means ± standard deviation (SD) and analyzed using SPSS 19.0 and GraphPad Prism 6.0 software (GraphPad Inc., San Diego, CA). Statistical analyses were conducted between two groups (control group vs. treatment groups, single treatment group vs. two treatments group) using a Student’s t-test. P values < 0.05 were considered to be statistically significant.

## Supplementary Information

**Additional file 1: Figure S1**. Selection of tumor cells and glucose concentrations. A) Tumor formation time in non-diabetic and diabetic mice implanted with four types of cancer cells. The formation time of the HT29 and HCT116 cells-formed tumors was the shortest one. Data are presented as mean ± SD, * *P* < 0.05 compared with the HT29 group (n = 5). B) Cell viabilities of HT29 cells in DMEM containing different concentrations of glucose (0, 1, 2, 3, 4, 5, 6, 7, 8 g·L^−1^). Data are presented as mean ± SD, * *P* < 0.05 compared with the control group (0 g·L^−1^) (n = 3). C) Cell apoptosis of HT29 cells in DMEM containing different concentrations of glucose (0, 1, 2, 3, 4, 5, 6 g·L^−1^). Data are presented as mean ± SD, * *P* < 0.05 compared with 1 g·L^−1^ glucose group or 6 g·L^−1^ glucose group (n = 3). D) Microscopy photographs (200 ×) showing LF inhibition of wound healing in HT29 cells cultured under both normal and high concentrations of glucose and the recovery rate of each scratch width under each treatment condition. Data are presented as mean ± SD, * *P* < 0.05 compared with control group, # *P* < 0.05 compared with high-glucose group (n = 3). At the same time-point of 24 h, the recovery of the scratch width of the high glucose (5 g·L^−1^)-cultured HT29 cells exceeded that in cells cultured under the normal condition (2 g·L^−1^), thus indicating that high glucose facilitated the migration of cancer cells. E) Microscopy photographs (200 ×) showing LF inhibition of the migration of HT29 cells under each treatment condition, and the quantification of migrated cells. Data are presented as mean ± SD, * *P* < 0.05 compared with control group, # *P* < 0.05 compared with high-glucose group (n = 3). LF (0.5 g·L^−1^) significantly suppressed cell migration under both culture conditions, indicating that the high glucose promoted the invasion of HT29 cancer cells, which could be notably mitigated by the LF.

**Additional file 2: Figure S2**. mRNA sequencing and DNA methylation detection. A) In mRNA sequencing, the functional classification of identified transcriptome-differentially expressed gene deep analysis (DEGs) by Gene Ontology database, through comparing normal colon epithelial cells (NCM460) and colon cancer cells (HT29). B) In mRNA sequencing, the KEGG pathway enrichment through comparing NCM460 cells and HT29 cells. C) Distribution map of differentially methylated loci, here, HT29 cells as the control group, HT29 cells treated with 0.5 g·L^−1^ lactoferrin in high glucose medium as the LF group (Supplementary DNA methylation profiling data in Data and Code Availability section) D) In methylation assay, the overview of differentially methylated genes (DMGs) in LF group, including 11,038 hyper-methylated genes and 9,595 hypo-methylated genes, when compared with HT29 control group. E) In methylation assay, the cluster heatmap of DMGs through comparing NCM460 cells and HT29 cells. F) In methylation assay, the DMG-related disease enrichment through comparing HT29 cells and the cells treated with LF. Based on the raw data analysis of Supplementary DNA methylation profiling data and results shown in (C-E), intriguingly, LF was found to cause a hypo-methylation of *NT5DC3*. These data indicate that *NT5DC3* is a bona fide downstream target of LF and suggest that *NT5DC3* might be a potential biomarker in colon tumor progression under hyperglycemia.

**Additional file 3: Figure S3**. The total 5mC/m^6^A and SAM/SAH ratio detected by MS, as well as the role of *WTAP* in regulating *NT5DC3* m^6^A. A) The levels of 5mC/C under different concentrations of glucose. B) The levels of m^6^A/A under different concentrations of glucose. C) The ratios of SAM/SAH under different concentrations of glucose. D) The normalized levels of *DNMT* with *NT5DC3* siRNA treatment. E) The levels of *NT5DC3* (5mC CpG island 1) with *NT5DC3* siRNA treatment. F) The normalized levels of *WTAP* with *NT5DC3* siRNA treatment. G) The levels of *NT5DC3* (m^6^A 2309) with *NT5DC3* siRNA treatment. H) The levels of *NT5DC3* (m^6^A 2309) with *WTAP* siRNA treatment. I) The levels of NT5DC3 and HKDC1 proteins with *WTAP* siRNA treatment. N stands for normal-glucose (2 g·L^−1^), H stands for high-glucose (5 g·L^−1^), LF stands for lactoferrin, H-N stands for the transfer from high-glucose to normal-glucose. The above data are presented as mean ± SD, * *P* < 0.05 compared with the control, & *P* < 0.05 compared with LF treatment group (n = 3).

**Additional file 4: Table S1.** Parameters in the 5mC/m^6^A detection by MS.

**Additional file 5: Table S2. **Sequence of primers used for qPCR, Select qPCR (m^6^A) and DNA methylation.

**Additional file 6: Table S3.** Characteristics of patients and healthy human subjects.

**Additional file 7: Table S4**. Type 2 diabetes indicators of mice.

## Data Availability

The ethics committee approval (Supplementary Ethics Committee approval), and the original/source data for transcriptomics analysis (Supplementary Transcriptomics analysis data.xls), DNA methylation profiling (Supplementary DNA methylation profiling data.txt) are available on request from the authors, which are from Mendeley Data: https://data.mendeley.com/datasets/gtsyfgk8rj/draft?a=da1d583d-72e1-4f0b-82cb-e88536d457d8.
