# Supplementary figures and images for: Lactoferrin suppresses the progression of colon cancer under hyperglycemia by targeting WTAP/m6A/NT5DC3/HKDC1 axis
Source: J Transl Med. 2023 Feb 28;21:156. doi: 10.1186/s12967-023-03983-1 (PMC9972781; doi:10.1186/s12967-023-03983-1)

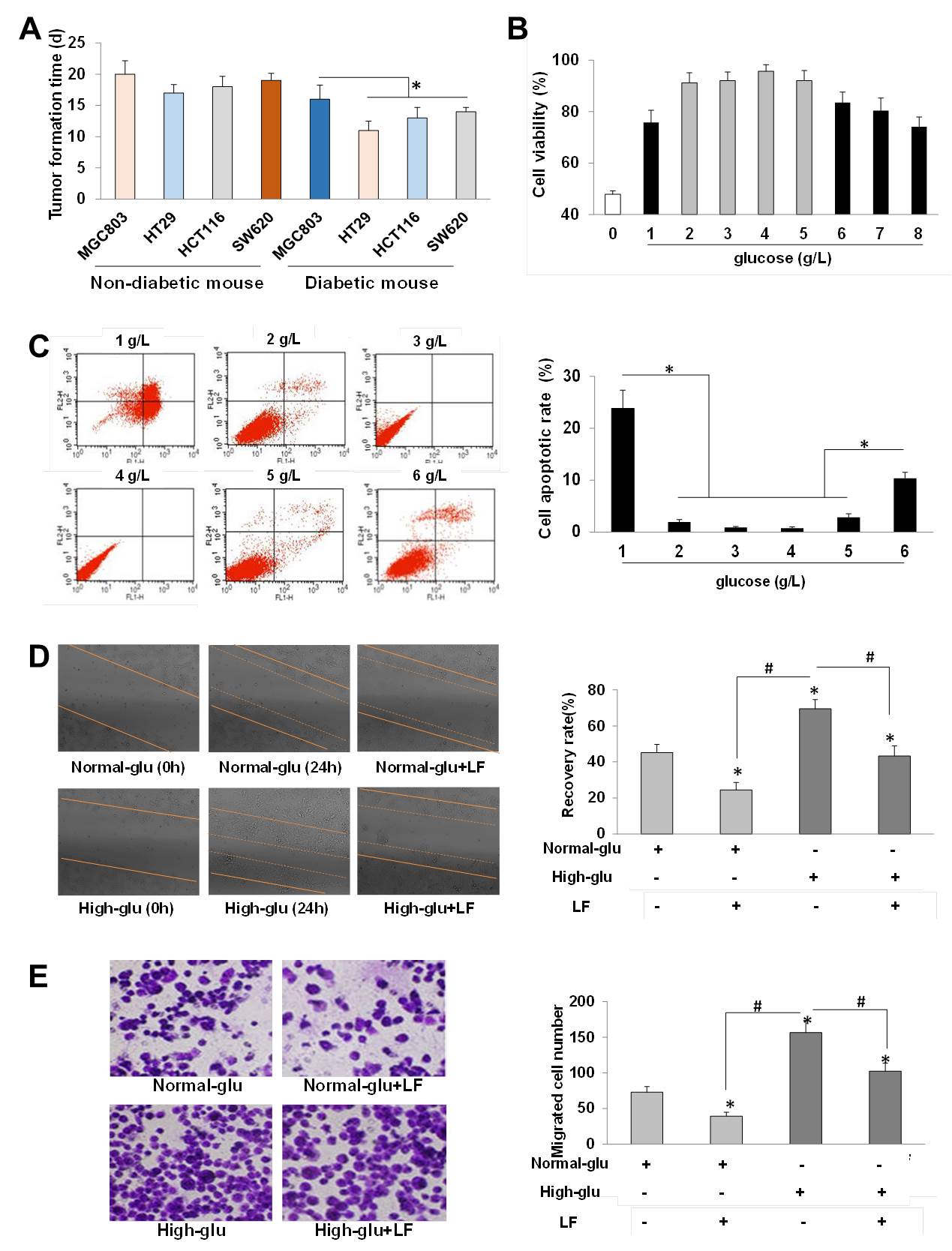

Supplement: Supplementary file 1 — Additional file 1: Figure S1. Selection of tumor cells and glucose concentrations. A) Tumor formation time in non-diabetic and diabetic mice implanted with four types of cancer cells. The formation time of the HT29 and HCT116 cells-formed tumors was the shortest one. Data are presented as mean ± SD, * P < 0.05 compared with the HT29 group (n = 5). B) Cell viabilities of HT29 cells in DMEM containing different concentrations of glucose (0, 1, 2, 3, 4, 5, 6, 7, 8 g·L−1). Data are presented as mean ± SD, * P < 0.05 compared with the control group (0 g·L−1) (n = 3). C) Cell apoptosis of HT29 cells in DMEM containing different concentrations of glucose (0, 1, 2, 3, 4, 5, 6 g·L−1). Data are presented as mean ± SD, * P < 0.05 compared with 1 g·L−1 glucose group or 6 g·L−1 glucose group (n = 3). D) Microscopy photographs (200 ×) showing LF inhibition of wound healing in HT29 cells cultured under both normal and high concentrations of glucose and the recovery rate of each scratch width under each treatment condition. Data are presented as mean ± SD, * P < 0.05 compared with control group, # P < 0.05 compared with high-glucose group (n = 3). At the same time-point of 24 h, the recovery of the scratch width of the high glucose (5 g·L−1)-cultured HT29 cells exceeded that in cells cultured under the normal condition (2 g·L−1), thus indicating that high glucose facilitated the migration of cancer cells. E) Microscopy photographs (200 ×) showing LF inhibition of the migration of HT29 cells under each treatment condition, and the quantification of migrated cells. Data are presented as mean ± SD, * P < 0.05 compared with control group, # P < 0.05 compared with high-glucose group (n = 3). LF (0.5 g·L−1) significantly suppressed cell migration under both culture conditions, indicating that the high glucose promoted the invasion of HT29 cancer cells, which could be notably mitigated by the LF. [file 12967_2023_3983_MOESM1_ESM.jpg]

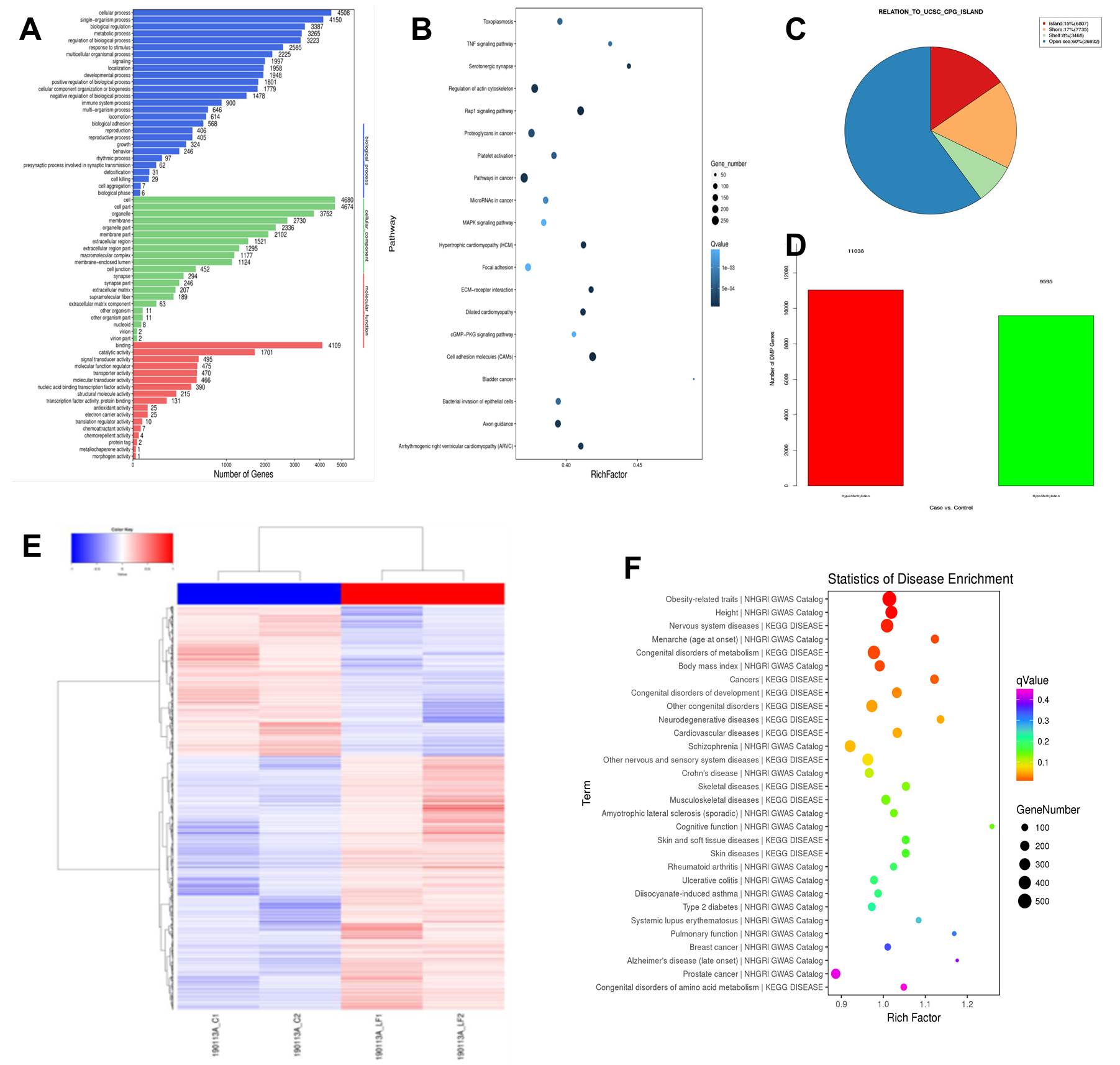

Supplement: Supplementary file 2 — Additional file 2: Figure S2. mRNA sequencing and DNA methylation detection. A) In mRNA sequencing, the functional classification of identified transcriptome-differentially expressed gene deep analysis (DEGs) by Gene Ontology database, through comparing normal colon epithelial cells (NCM460) and colon cancer cells (HT29). B) In mRNA sequencing, the KEGG pathway enrichment through comparing NCM460 cells and HT29 cells. C) Distribution map of differentially methylated loci, here, HT29 cells as the control group, HT29 cells treated with 0.5 g·L−1 lactoferrin in high glucose medium as the LF group (Supplementary DNA methylation profiling data in Data and Code Availability section) D) In methylation assay, the overview of differentially methylated genes (DMGs) in LF group, including 11,038 hyper-methylated genes and 9,595 hypo-methylated genes, when compared with HT29 control group. E) In methylation assay, the cluster heatmap of DMGs through comparing NCM460 cells and HT29 cells. F) In methylation assay, the DMG-related disease enrichment through comparing HT29 cells and the cells treated with LF. Based on the raw data analysis of Supplementary DNA methylation profiling data and results shown in (C-E), intriguingly, LF was found to cause a hypo-methylation of NT5DC3. These data indicate that NT5DC3 is a bona fide downstream target of LF and suggest that NT5DC3 might be a potential biomarker in colon tumor progression under hyperglycemia. [file 12967_2023_3983_MOESM2_ESM.jpg]

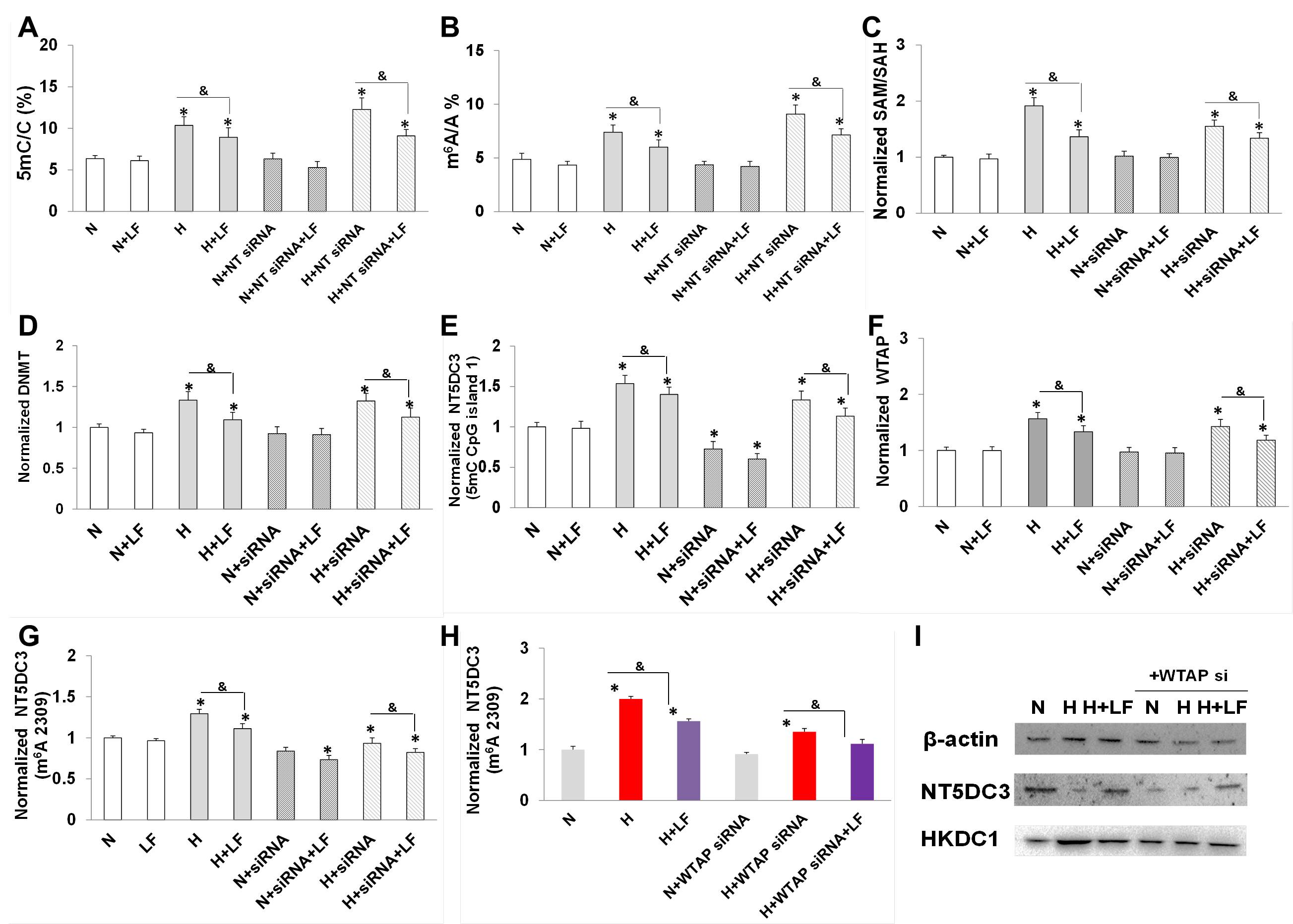

Supplement: Supplementary file 3 — Additional file 3: Figure S3. The total 5mC/m6A and SAM/SAH ratio detected by MS, as well as the role of WTAP in regulating NT5DC3 m6A. A) The levels of 5mC/C under different concentrations of glucose. B) The levels of m6A/A under different concentrations of glucose. C) The ratios of SAM/SAH under different concentrations of glucose. D) The normalized levels of DNMT with NT5DC3 siRNA treatment. E) The levels of NT5DC3 (5mC CpG island 1) with NT5DC3 siRNA treatment. F) The normalized levels of WTAP with NT5DC3 siRNA treatment. G) The levels of NT5DC3 (m6A 2309) with NT5DC3 siRNA treatment. H) The levels of NT5DC3 (m6A 2309) with WTAP siRNA treatment. I) The levels of NT5DC3 and HKDC1 proteins with WTAP siRNA treatment. N stands for normal-glucose (2 g·L−1), H stands for high-glucose (5 g·L−1), LF stands for lactoferrin, H-N stands for the transfer from high-glucose to normal-glucose. The above data are presented as mean ± SD, * P < 0.05 compared with the control, & P < 0.05 compared with LF treatment group (n = 3). [file 12967_2023_3983_MOESM3_ESM.jpg]
