# Supplementary material for: Lactoferrin suppresses the progression of colon cancer under hyperglycemia by targeting WTAP/m6A/NT5DC3/HKDC1 axis
Source: J Transl Med. 2023 Feb 28;21:156. doi: 10.1186/s12967-023-03983-1 (PMC9972781; doi:10.1186/s12967-023-03983-1)
Supplement: Supplementary file 4 — Additional file 4: Table S1. Parameters in the 5mC/m6A detection by MS. [file 12967_2023_3983_MOESM4_ESM.docx]

**Table S1. Parameters in the 5mC/m^6^A detection by MS**

| Compound | Parent (*m/z*) | Daughters  (*m/z*) | Cone Voltage  (V) | Collision Energy  (V) | Ion Mode | Comments |
| --- | --- | --- | --- | --- | --- | --- |
| Adenosine | 268.23 | 136.12 | 22 | 32 | ES+ | Quantitative ion pair |
|  | 268.23 | 119.09 | 22 | 42 | ES+ | Qualitative ion pair |
| m^6^A | 282.3 | 150.15 | 8 | 18 | ES+ | Quantitative ion pair |
|  | 282.3 | 108.09 | 8 | 62 | ES+ | Qualitative ion pair |
| Cytidine | 244.23 | 112.06 | 28 | 10 | ES+ | Quantitative ion pair |
|  | 244.23 | 94.96 | 28 | 46 | ES+ | Qualitative ion pair |
| 5mC | 258.23 | 126.11 | 22 | 10 | ES+ | Quantitative ion pair |
|  | 258.23 | 108.89 | 22 | 42 | ES+ | Qualitative ion pair |
| Thymidine (IS) | 243.23 | 127.11 | 16 | 10 | ES+ | Quantitative ion pair |
|  | 243.23 | 117.06 | 16 | 6 | ES+ | Qualitative ion pair |
