# Supplementary material for: Lactoferrin suppresses the progression of colon cancer under hyperglycemia by targeting WTAP/m6A/NT5DC3/HKDC1 axis
Source: J Transl Med. 2023 Feb 28;21:156. doi: 10.1186/s12967-023-03983-1 (PMC9972781; doi:10.1186/s12967-023-03983-1)
Supplement: Supplementary file 5 — Additional file 5: Table S2. Sequence of primers used for qPCR, Select qPCR (m6A) and DNA methylation. [file 12967_2023_3983_MOESM5_ESM.docx]

**Table S2.** **Sequence of primers used for qPCR, Select qPCR (m^6^A) and DNA methylation**

| Gene name | Primer Sequences (5' → 3') | |
| --- | --- | --- |
|  | Forward primer | Reverse primer |
| *NT5DC3* | CACAGAGATGCTGAGTCACAGC | GAACAGGCTTCCAAACTGGGCA |
| *HKDC1* | ATCGCCGACTTCCTGGACTACA | GCCTTGAAACCTTTGGTCCACC |
| *NT5DC3-*X | tagccagtaccgtagtgcgtgCTCTTGTTCTTTCTCACTTAAG | 5phos/CCTTCCAGGGAGGCTGTGTTCcagaggctgagtcgctgcat |
| *NT5DC3-*N | tagccagtaccgtagtgcgtgGTCCTTCCAGGGAGGC | 5phos/GTGTTCTATTTGATGTGATGcagaggctgagtcgctgcat |
| *GAPDH-*X | tagccagtaccgtagtgcgtgCAGTAGAGGCAGGGATGATG | 5phos/TCTGGAGAGCCCCGCGGCCAcagaggctgagtcgctgcat |
| *GAPDH-*N | tagccagtaccgtagtgcgtgTTCTGGAGAGCCCCGCGGCCA | 5phos/CACGCCACAGTTTCCCGGAcagaggctgagtcgctgcat |
| *NT5DC3* select | ATGCAGCGACTCAGCCTCTG | TAGCCAGTACCGTAGTGCGTG |
| *METTL3* | ATCCCCAAGGCTTCAACCAG | GCGAGTGCCAGGAGATAGTC |
| *METTL14* | AGAGAACAAAGGAACACTGCCT | AATGAAGTCCCCGTCTGTGC |
| *WTAP* | CAGCTGCTCCATTGTGCCT | TCACTCAATCGAACCTTCTTGG |
| *FTO* | GGTCGAGTTTGAGTGGCTGA | GCATTTGTTGCCTTGTGCCT |
| *ALKBH5* | TGACTGTGCTCAGTGGATATG | TGACAGGCGATCTGAAGCAT |
| *YTHDF1* | CGTGGACACCCAGAGAACAA | TAGCTGGACAGGTAGGGGTC |
| *YTHDF2* | GTCAGGGACAAAAGCCTCCG | GACCTTTTGGTCTCTGCTCCA |
| *YTHDF3* | ATTGTGGACCCGAGAAGCAG | CCTTGCCCTTTAGGTCTCTGA |
| *DNMT* | GGCGGCTCAAAGATTTGGAA | CAGGTAGCCCTCCTCGGAT |
| *NT5DC3* m2 | CAGGCAGCAGGCATGACCAT | TTCTGTGCTTCTCTTCGCCTCC |
| *GAPDH* MSPM | CATGGCACCGTCAAGGCTGAGAACGG | GCCTTCTCCATGGTGGTGAAGACGCC |
| siRNA-*NT5DC3* | GAGAAAUGACCAAGAGUUUTT | AAACUCUUGGUCAUUUCUCGC |
| siRNA-NC | UUCUCCGAACGUGUCACGGUTT | ACGUGACACGUUCGGAGAATT |
| siRNA-*METTL3* | GCTGCACTTCAGACGAATTAT | ATAATTCGTCTGAAGTGCAGC |
| siRNA-*METTL14* | GCCGTGGACGAGAAAGAAATA | TATTTCTTTCTCGTCCACGGC |
| siRNA-*WTAP* | GCAAGAGTGTACTACTCAAAT | ATTTGAGTAGTACACTCTTGC |
| siRNA-*FTO* | CAACGTAACTTTGCTGAATTT | AAATTCAGCAAAGTTACGTTG |
| siRNA-*ALKBH5* | CCACCCAGCTATGCTTCAGAT | ATCTGAAGCATAGCTGGGTGG |
| siRNA-*YTHDF1* | ACGACATCCACCGCTCCATTA | TAATGGAGCGGTGGATGTCGT |
| siRNA-*YTHDF2* | CGGTCCATTAATAACTATAAC | GTTATAGTTATTAATGGACCG |
| siRNA-*YTHDF3* | GCTACTCTGAGGATGACATAC | GTATGTCATCCTCAGAGTAGC |
