# Supplementary material for: Lactoferrin suppresses the progression of colon cancer under hyperglycemia by targeting WTAP/m6A/NT5DC3/HKDC1 axis
Source: J Transl Med. 2023 Feb 28;21:156. doi: 10.1186/s12967-023-03983-1 (PMC9972781; doi:10.1186/s12967-023-03983-1)
Supplement: Supplementary file 6 — Additional file 6: Table S3. Characteristics of patients and healthy human subjects. [file 12967_2023_3983_MOESM6_ESM.docx]

**Table S3. Characteristics of patients and healthy human subjects**

|  | Healthy | Type 2 diabetes(T2D) | T2D-induced colon cancer |
| --- | --- | --- | --- |
| Number | 30 | 30 | 15 |
| Age | 58.6±9.9 | 60.7±7.7 | 61.5±5.2 |
| Sex(male/female) | 15/15 | 15/15 | 8/7 |
| Average of fasting blood glucose (mmol·L^-1^) | 4.9±1.1 | 10.5±3.7 | 10.5±3.7 |
| Diagnose | Healthy | Type 2 diabetes | T2D and colon cancer (later) |
