# Supplementary material for: Lactoferrin suppresses the progression of colon cancer under hyperglycemia by targeting WTAP/m6A/NT5DC3/HKDC1 axis
Source: J Transl Med. 2023 Feb 28;21:156. doi: 10.1186/s12967-023-03983-1 (PMC9972781; doi:10.1186/s12967-023-03983-1)
Supplement: Supplementary file 7 — Additional file 7: Table S4. Type 2 diabetes indicators of mice. [file 12967_2023_3983_MOESM7_ESM.docx]

**Table S4. Type 2 diabetes indicators of mice**

| indicators | healthy mice | T2D mice |
| --- | --- | --- |
| fasting blood glucose detection (FBG, mM) | 4.2±0.6 | 27.8±3.4 |
| oral glucose tolerance test (OGTT, mM) | 8.1±1.1 | 25.0± 5.3 |
| glycated serum protein (GSP, mM) | 1.8±0.1 | 2.6±0.7 |
| serum insulin (INS, mIU/L) | 61.1±4.7 | 33.8±4.3 |
